# In the Age of Artificial Intelligence: From Standardized Questions to Real-World Breastfeeding Concerns

**DOI:** 10.3390/jcm15197513

**Published:** 2026-09-27

**Authors:** Maria Kkirgia, Elli-Panagiota Magklara, Christiana Zoupa, Paraskevi Volaki, Vasiliki Mougiou, Zoi Iliodromiti, Theodora Boutsikou, Nicoletta Iacovidou, Rozeta Sokou

**Affiliations:** 1 Neonatal Department, Aretaieio Hospital, National and Kapodistrian University of Athens, 11528 Athens, Greece; kirgiamaria@icloud.com (M.K.); ellimaglara99@gmail.com (E.-P.M.); v.volaki@hotmail.com (P.V.); vasiamoug@med.uoa.gr (V.M.); ziliodromiti@med.uoa.gr (Z.I.); theobtsk@med.uoa.gr (T.B.); niakobid@med.uoa.gr (N.I.); 2 Artificial Intelligence for Business, Bologna Business School, 40136 Bologna, Italy; christiana.zoupa@bbscommunity.it

**Keywords:** Artificial Intelligence, breastfeeding, large language models, lactation support, neonatal support, postpartum care

## Abstract

**Background**: Breastfeeding is the recommended source of infant nutrition, yet mothers may experience concerns and difficulties that require timely access to reliable information and professional support. As the use of digital health tools increases, AI-powered chatbots such as ChatGPT and Gemini are increasingly accessible sources of breastfeeding information and support. This study aimed to descriptively compare responses generated by ChatGPT and Gemini with established breastfeeding information and multidisciplinary clinical support provided in real-world practice, using both standardized breastfeeding questions and clinic-derived scenarios. **Materials and Methods:** A descriptive comparative content-analysis study was conducted at Aretaieio University Hospital. Ten breastfeeding-related questions were included: five standardized questions derived from educational materials of the Institute of Child Health in Greece and five clinic-derived scenarios originating from individualized consultations through the hospital’s breastfeeding-support helpline. Identical prompts were submitted independently to ChatGPT and Gemini as single-turn interactions without supplementary information or follow-up. AI-generated responses to the five standardized questions were descriptively compared with the corresponding information provided by the Institute of Child Health, whereas responses to five clinic-derived scenarios were compared with the clinical guidance and management provided in real-world practice. **Results**: Both AI platforms provided relevant, detailed, and supportive responses, particularly to standardized questions. However, the clinic-derived scenarios highlighted dimensions of individualized lactation care that extended beyond the AI-generated responses, particularly direct clinical assessment, access to relevant clinical history, interpretation of emotional and contextual cues, continuity of care, and coordination of multidisciplinary care and practical support. **Conclusions**: As AI becomes increasingly integrated into everyday health information-seeking, its role in breastfeeding support needs to be clearly defined. AI platforms may complement professional care by providing accessible preliminary information and support, while individualized breastfeeding care remains rooted in direct assessment, continuity of professional care, and trusted human relationships.

## 1. Introduction

It is widely accepted that breast milk is the optimal source of nutrition for the neonate and the infant, conferring substantial short- and long-term benefits on both the mother and infant [1]. Global health authorities, specifically the World Health Organization (WHO) and UNICEF, advise that breastfeeding should commence within the first hour after birth. To ensure optimal development, infants should be exclusively breastfed for the first six months, without receiving any other foods or liquids, including water. Thereafter, breastfeeding should continue alongside the introduction of appropriate complementary foods until two years of age. [2]. A WHO/UNICEF Discussion Paper on extending the Global Nutrition Targets to 2030 proposed a target of at least 70% exclusive breastfeeding during the first six months of life [3]. However, in 2025, the 78th World Health Assembly adopted a revised global target of at least 60% by 2030 [4].

Globally, exclusive breastfeeding has increased gradually, with the proportion of infants younger than six months who were exclusively breastfed rising from 40.7% in 2012 to 45.1% in 2021 and approaching 48% in subsequent years, coming close to the 2025 milestone of 50% [5]. Despite this progress, rates remain below international targets, and further progress will be needed to meet the higher target set for 2030 [4]. The WHO European Region has the lowest rates worldwide, with approximately 25% of infants exclusively breastfed at six months; among high-income countries, the median rate at six months is only 18% [6]. In Greece, despite high rates of breastfeeding initiation, maintaining exclusive breastfeeding remains a considerable challenge. National data demonstrate a marked disparity between initiation and continuation: in 2017, although 94% of mothers initiated breastfeeding, the proportion exclusively breastfeeding fell to approximately 40% at one month, 25% at four months and less than 1% at six months [7]. Breastfeeding-supportive practices in maternity facilities also remained suboptimal: rooming-in (63%), skin-to-skin contact within the first hour (51%), and the provision of free formula samples at discharge (19%). Data from Cyprus similarly indicate a disparity between intention and early practice: although 81.9% of mothers reported an intention to breastfeed exclusively, 18.8% were doing so at 48 h [8]. These findings highlight the importance of distinguishing breastfeeding initiation, continuation of any breastfeeding, and maintenance of exclusive breastfeeding through six months. Early discontinuation of exclusive breastfeeding is associated with multiple maternal, infant, social, and environmental factors [6,9,10,11,12]. Perceived insufficient milk supply, nipple pain and other breastfeeding difficulties, as well as maternal fatigue are among the most frequently reported reasons for early discontinuation [13,14,15]. Lower maternal breastfeeding self-efficacy and limited or delayed access to specialized professional support may also affect breastfeeding exclusivity and duration [7,8,10,12,16]. Family and social circumstances, cultural beliefs, support from partners and relatives, and employment-related factors may further influence these outcomes [17,18]. In addition, maternal chronic illness, infant illness or hospitalization, and the resulting separation of mother and infant may impede the establishment and maintenance of exclusive breastfeeding [13,15,19,20]. These factors correspond to many of the concerns for which mothers seek support during lactation. Timely access to specialized professional support may help address these difficulties and sustain breastfeeding.

Midwives play a vital role in supporting and promoting breastfeeding by providing clinical guidance, maternal education and psychosocial support. Nevertheless, the availability of midwifery care is often constrained by geographic, financial, or structural barriers [21]. Maternal concerns related to lactation, such as perceived milk insufficiency, remain a primary factor contributing to early breastfeeding discontinuation [22]. Driven by these difficulties, mothers often utilize online platforms and digital technologies to access quick, individualized advice and support concerning lactation, newborn health, and postpartum maternal well-being. More recently, AI chatbots, including ChatGPT and Google Gemini, have provided the public with easily accessible tools for generating on-demand medical content [23].

AI technologies can simulate aspects of human interaction, address complex queries, and generate decision-support information. [24]. However, significant doubts about their trustworthiness remain. These include the potential dissemination of misinformation, the promotion of non-evidence-based therapies and inadequate communication of the risks associated with conventional medical treatments [25]. At the same time, a further limitation of AI in healthcare is its inability to replicate the empathetic connection typically formed between a medical practitioner (for example a midwife/doctor) and a patient (for example a mother) [26]. Given the increasing use of digital tools, it is crucial to assess whether chatbot-generated breastfeeding advice is consistent with established clinical guidelines and scientific evidence.

AI-assisted breastfeeding counseling has demonstrated promising effects on maternal breastfeeding self-efficacy and breastfeeding success [25], while an AI-powered SMS intervention was perceived as useful and usable by postpartum mothers, although limitations in comprehension and human-like interaction remained [27]. Chatbots have also been developed to provide accessible emotional and mental-health support during pregnancy and the postpartum period [28]. The quality of AI-generated maternal-health information has been investigated in several clinical contexts. ChatGPT has demonstrated adequate performance in answering questions about puerperal mastitis, although variability between professional evaluators and concerns regarding reliability and accessibility were reported [29].

Evaluations of pregnancy-related oral-health advice similarly found that ChatGPT could provide useful information but that its responses required revision and supervision by healthcare professionals [30]. In postpartum discharge education, AI produced more comprehensive general information than nurses, whereas human input remained essential for individualized and practical recommendations [31]. Most directly relevant to breastfeeding, Kacer [32] evaluated ChatGPT, Gemini and Copilot (Microsoft Edge, 2023) using 50 frequently asked breastfeeding questions and reported generally high quality and reliability; however, the responses required a relatively high educational reading level, raising concerns about their accessibility to all mothers.

Nevertheless, previous studies have primarily assessed standardized informational questions, individual clinical conditions or purpose-built digital interventions. The ability of contemporary general-purpose LLMs to respond to complex breastfeeding concerns that involve emotional distress, socioeconomic barriers, sensitive sexual experiences, continuity of care and the need for clinical verification remains insufficiently characterized. Furthermore, limited research has directly compared AI-generated responses with the support provided by a hospital-based multidisciplinary breastfeeding team. Such support extends beyond the provision of information to include clinical assessment, access to medical records, coordination with other healthcare professionals, mobilization of practical resources, professional boundary-setting and longitudinal follow-up. Breastfeeding mothers frequently seek timely information, practical guidance, and reassurance regarding concerns arising during lactation, particularly to determine whether breastfeeding is progressing effectively and whether their infant is receiving adequate nutrition.

In this context, the present study aimed to descriptively compare the content and characteristics of responses generated by two widely available large language model (LLM)-based platforms, ChatGPT and Gemini, with established breastfeeding information and real-world multidisciplinary clinical support. The comparison included ten breastfeeding-related prompts representing two levels of complexity: five standardized questions derived from educational materials of the Institute of Child Health in Greece (https://eclass.epilegothilasmo.gr/) and five clinic-derived scenarios reflecting concerns encountered through the hospital’s specialized breastfeeding-support helpline. Attention was given to areas of convergence and divergence in the information and practical guidance provided, safety-related advice and escalation, emotional reassurance, and the extent to which responses addressed the contextual and individualized aspects of breastfeeding concerns.

## 2. Methods

The Neonatal and Obstetric Clinic of our hospital is recognized as a ‘Baby-Friendly Hospital’ in accordance with UNICEF standards for the promotion and support of breastfeeding [33]. The hospital also operates a 24 h breastfeeding-support helpline staffed by trained midwives working in collaboration with pediatricians and neonatologists, providing guidance and support to pregnant and postpartum women.

A descriptive comparative content-analysis study was conducted to explore the characteristics of breastfeeding-related responses generated by two publicly available LLM-based platforms, ChatGPT (OpenAI) and Gemini (Google), in relation to established breastfeeding information and real-world multidisciplinary clinical support. Hereafter, the two systems are referred to collectively as “AI chatbots” or “AI platforms”.

Ten breastfeeding-related prompts were selected from two sources to represent different levels of clinical and contextual complexity.

Five standardized questions were derived from educational materials and frequently asked questions published by the Institute of Child Health in Greece [34].

The five clinic-derived scenarios were purposively selected from mothers’ questions documented by the hospital’s 24 h breastfeeding-support helpline between 3 May and 25 July 2026. The selection aimed to include actual clinical concerns that differed from the five standardized questions published by the Institute of Child Health and required more individualized consideration of the clinical, psychosocial, or practical context. The scenarios were based on documentation recorded at the time of the consultation rather than retrospective recall or composite cases. As part of routine helpline practice, staff document the consultation, the guidance provided, and, when indicated, subsequent management and follow-up. At the start of a consultation, mothers are asked whether they wish to provide their name and contact details solely to facilitate continuity of support and follow-up when needed. For this study, no direct identifiers, including names or telephone numbers, were extracted from the records, used in the analysis, or included in the scenarios or prompts submitted to the AI platforms. The clinic-derived scenarios were used only in de-identified form; only the clinical and contextual information necessary to represent each concern and conduct the comparison was retained.

The source used for descriptive comparison differed according to the type of prompt. For the five standardized questions originating from the Institute of Child Health, the corresponding answers provided by the Institute, which had been developed through expert consensus, were used as the reference standard. For the five clinic-derived scenarios, AI-generated responses were compared with the documented guidance and management provided in routine clinical practice by experienced midwives specializing in breastfeeding support and lactation counseling, in collaboration with neonatologists. These clinical responses could include targeted history-taking, individualized counseling, review of relevant clinical information, direct assessment when indicated, follow-up, and coordination with other healthcare professionals or support resources, depending on the circumstances of each case.

Three reviewers (R.S., Z.I. and T.B) independently conducted the comparative assessment. All three are professors of pediatrics–neonatology, each with more than 10 years of clinical experience in breastfeeding support at a Baby-Friendly Hospital. Each reviewer independently assessed every response against the predefined comparison domains. Differences in interpretation were discussed to reach consensus. If consensus could not be reached, assessment by a fourth senior reviewer (N.I.; a professor of pediatrics-neonatology with more than 30 years of clinical experience in neonatology and more than 10 years of experience in breastfeeding support at a Baby-Friendly Hospital.) was prespecified.

The comparison domains were defined a priori based on the study aim and the principal dimensions of breastfeeding support. Five domains were assessed: (i) informational content and clinical accuracy, including points of agreement, omissions, and inaccuracies; (ii) practical guidance provided to the mother; (iii) recognition of potential warning signs, safety-related guidance, and recommendations to seek professional or clinical assessment when indicated; (iv) supportive and empathetic communication and emotional reassurance; and (v) the extent to which the response considered the individual clinical, psychosocial, and practical context of the concern. Each response generated by the two AI platforms was examined descriptively across the same predefined domains and compared with the corresponding reference source: the Institute of Child Health answers for the five standardized questions and the support and management provided by the multidisciplinary clinical team for the five clinic-derived scenarios. No numerical scoring system, weighted assessment, or overall ranking of responses was applied.

Each of the ten prompts was submitted separately to ChatGPT and Gemini using identical wording. All prompts were submitted in English. For the five standardized questions, we used the official English version of “Frequently Asked Questions About Breasfeeding,” published by the Institute of Child Health’s National Initiative for the Promotion of Breastfeeding, “ALKYONI” [34]. The clinic-derived scenarios originated from telephone consultations conducted in Greek and were translated into English by N.I.

Each question was entered as a new single, stand-alone prompt without supplementary instructions or prior conversational context. No follow-up questions, additional contextual information, rephrasing, or regeneration of responses were permitted. This single-turn approach was selected to examine the type of immediate response available to a mother entering an isolated breastfeeding-related concern into a general-purpose AI platform. Both platforms were accessed through their web interfaces. Neither platform was explicitly instructed to conduct a web search, and no separate deep research or research mode was used. Gemini’s integrated Google Search functionality was not disabled; therefore, the possibility that its responses incorporated information retrieved through that functionality cannot be excluded. The AI responses were generated on 5 and 26 July 2026.

Unlike the standardized questions, the clinic-derived scenarios arose from individualized telephone consultations conducted through the hospital’s breastfeeding-support helpline. At the beginning of each consultation, the midwife introduced herself and invited the caller to provide her name and contact details, allowing further communication if the mother wished. Targeted questions were used to assess both the reported breastfeeding concern and the mother’s broader clinical and psychosocial circumstances. Attention was also given to paralinguistic cues, including tone of voice, speech volume and pace, hesitation, and crying, which could prompt further sensitive questioning.

The nature and extent of each consultation were guided by the mother’s individual needs. Some mothers contacted the service with a specific breastfeeding problem, whereas others contacted the service without a specific clinical concern, primarily seeking reassurance that breastfeeding was progressing appropriately or wishing to speak with a healthcare professional. The duration and scope of these consultations therefore varied according to the nature and complexity of the concern and were documented when relevant.

Mothers frequently described distinctive characteristics of their infants, such as their appearance or behavior, which could help the midwife recall the family and identify the particular infant. This often reflected an already established relationship between the family and the service. When relevant and available, maternal and infant medical records, together with documentation of previous contacts with the service, were reviewed to provide additional clinical context. The helpline also allowed continuity of support beyond the initial telephone contact. Mothers were encouraged to contact the service again whenever further concerns arose. With their permission, follow-up was usually undertaken by the same midwife who had provided the initial guidance and support. Mothers also frequently requested to speak again with the midwife with whom they had first communicated. Depending on the nature of the concern, support could extend beyond telephone counseling to direct clinical assessment, collaboration with neonatologists or other healthcare professionals, and practical interventions or referral when required. Thus, the clinic-derived responses reflected an evolving, context-informed process of breastfeeding support rather than a single, isolated response to the initial concern.

The complete AI-generated responses were retained and are presented verbatim to preserve the original output and enable direct examination of the breadth, structure, and nature of the information provided by each platform.

## 3. Results

### 3.1. Standardized Breastfeeding Questions

The first part of the descriptive comparison included five standardized breastfeeding questions derived from educational materials and frequently asked questions published by the Institute of Child Health in Greece. Each question was submitted independently to ChatGPT and Gemini, generating ten AI responses. The responses were examined in relation to the corresponding information provided by the Institute of Child Health, with attention to informational content, practical guidance, safety-related advice and escalation, emotional reassurance, and contextualization. The complete AI-generated responses and the principal areas of convergence and divergence are presented in Table S1.

#### Overall Observations from Standardized Questions

Across the five standardized questions, both AI platforms addressed the principal breastfeeding concerns raised in the prompts and were broadly consistent with the information provided by the Institute of Child Health. The AI-generated responses were generally more extensive than the corresponding Institute information and frequently supplemented the core information with practical suggestions, warning signs, and recommendations regarding when professional assessment should be sought (Table 1).

For concerns regarding milk sufficiency, both platforms emphasized observable indicators such as infant weight gain, wet and soiled diaper output, feeding behavior, and signs of effective milk transfer. They also distinguished common maternal concerns, such as frequent feeding or low expressed milk volume, from more clinically relevant indicators of inadequate intake. For the question concerning diaper output, both platforms provided age-related expectations and identified reduced urine output as a potential indication for further assessment. Similarly, in response to the sleepy newborn scenario, both platforms considered infant age, weight trajectory, feeding frequency, diaper output, and clinical condition, and included recommendations for seeking medical assessment when persistent lethargy or difficulty waking was present.

For breast redness and pain, both platforms discussed mastitis or inflammatory breast conditions, continuation of breastfeeding or milk expression, symptom management, and circumstances requiring medical evaluation. However, several points were identified that require clinical interpretation within the contemporary mastitis spectrum model. Specifically, recommendations for frequent ‘’emptying’’ of the affected breast, require careful interpretation, as excessive milk removal may exacerbate milk oversupply and inflammation. In addition, Gemini’s statement that mastitis generally requires antibiotic treatment does not adequately distinguish between inflammatory and bacterial mastitis, as antibiotic therapy is not indicated in all cases [35]. For breast refusal, the responses considered multiple potential contributing factors and proposed practical strategies such as skin-to-skin contact, changes in feeding position or environment, protection of maternal milk supply, and professional assessment when feeding difficulties persisted or signs of inadequate intake were present.

In addition to informational and practical content, both platforms frequently incorporated reassuring or empathetic language and often invited the mother to provide further information that could allow a more individualized response. However, because each prompt was examined as a single-turn interaction, these requests for additional information were not pursued further.

### 3.2. Clinic-Derived Breastfeeding Scenarios

The second part of the descriptive comparison included five breastfeeding concerns derived from individualized consultations with mothers contacting the hospital’s breastfeeding-support helpline. Each scenario was submitted independently to ChatGPT and Gemini, generating ten AI responses. These responses were examined in relation to the guidance and management provided by the multidisciplinary clinical team during the corresponding real-world consultations. The scenarios, clinical responses, AI-generated responses, and principal comparative observations are presented in Table S2.

#### Overall Observations from Clinic-Derived Scenarios

Across the five clinic-derived scenarios, both AI platforms provided informational and practical guidance and frequently incorporated reassuring or supportive language. Their responses also commonly included recommendations for professional assessment or additional support and, in several cases, requests for further information that could allow more individualized guidance (Table 2).

In the descriptive evaluation of clinical accuracy, specific areas requiring clarification were also identified. In the scenario concerning aversion to breast touch, Gemini’s verbatim response used the term “Dysmorphic Milk Ejection Reflex”, whereas the established term is “Dysphoric Milk Ejection Reflex (D-MER)”. The original phrasing was kept unchanged in the full AI-generated output in the Supplementary Material, while the terminological inaccuracy is highlighted in the descriptive comparative analysis. Furthermore, the reference to D-MER as a potential explanation requires clinical interpretation, as D-MER is characterized by transient onset of negative emotions temporally related to the milk-ejection reflex and is not necessarily identical to generalized aversion to breast touch [36,37].

In contrast to the single-turn AI responses, the clinical consultations evolved through targeted questioning and the incorporation of additional information regarding the mother, infant, feeding history, and wider circumstances. In the individual clinical scenarios, the additional information that emerged during the conversation was about, among other things, maternal exhaustion and emotional distress, specific breastfeeding practices, the objective assessment of the infant’s weight gain, and practical or financial limitations affecting the feasibility of the proposed interventions. Where required, clinical management included direct examination and weighing of the infant, communication with the attending pediatrician, arrangement of psychological support, practical facilitation of access to breastfeeding equipment and follow-up evaluation. Paralinguistic and emotional cues, previous interactions with the service, and available clinical information also contributed to the guidance provided during the consultations.

## 4. Discussion

The present study compared responses generated by ChatGPT and Gemini with established breastfeeding information from the Greek Institute of Child Health and with real-world guidance and management provided by an experienced multidisciplinary breastfeeding team at our hospital. Across the standardized questions, both AI platforms successfully produced pertinent, detailed, and supportive guidance, with substantial overlap with the established breastfeeding information. In contrast, the clinic-derived scenarios highlighted that lactation support necessitates far more than mere information transfer. Crucial dimensions of care, including physical assessment, access to longitudinal medical history, interpretation of paralinguistic and emotional cues, and the coordination of professional resources to navigate socioeconomic barriers, were available within the clinical setting but extended beyond the scope of the isolated AI responses examined in this study.

Our observations align with existing literature suggesting that AI-mediated health content is often perceived as comprehensive and empathetic yet still requires professional oversight. In an analysis of patient inquiries on social media, Ayers et al. reported that chatbot responses were favored over those from physicians, receiving superior marks for both quality and empathy. However, these AI-generated replies were notably more verbose, and the study focused on textual quality rather than diagnostic precision or longitudinal clinical outcomes [38].

In a systematic review of 20 studies comparing LLM-generated responses with those of healthcare professionals or clinical guidelines, Jacobs et al. found that 12 studies favored LLM responses, six reported mixed findings, and two favored healthcare professionals. The authors concluded that LLMs should currently be considered supportive tools rather than substitutes for professional expertise [39].

In contrast to the generally favorable findings reported in some studies examining the use of large language models in healthcare, Meyer et al. identified important limitations in the interpretation of medical laboratory results by AI-based chatbots [40]. Their study compared three AI chatbots- ChatGPT, Gemini, and Le Chat- with physician responses to 100 real patient questions concerning complete blood count results. In general, the chatbots demonstrated lower accuracy than physicians, with their responses frequently containing incorrect or overly generalized interpretations. However, they were perceived as more empathetic in their communication [40]. Taken together, these findings highlight an important distinction between the characteristics of an AI-generated textual response and the broader process of clinical care, which may additionally involve contextual assessment, verification, follow-up, and clinical intervention.

The findings of the present study highlight the need to distinguish between different dimensions of the quality of an AI-generated response. Informational completeness concerns the range and detail of provided information, while clinical accuracy concerns whether the specific information and recommendations are medically correct and appropriate for the respective clinical context. Supportive or empathetic language constitutes a distinct dimension and does not in itself either clinical accuracy or personalized care. Finally, practical care delivery includes capabilities such as direct clinical evaluation, verification of findings, coordination with other healthcare providers, addressing practical barriers and follow-up, which were not evaluated as capabilities of AI platforms in the single-turn design of the present study.

Specific to lactation, Kacer found generally high reliability across major AI platforms for common breastfeeding questions [32]. Nevertheless, the high educational reading level required for these responses raised accessibility concerns. Our study extends this research beyond standardized breastfeeding questions by also examining clinic-derived scenarios originating from real-world consultations and involving emotional distress, socioeconomic obstacles, individualized assessment, and the need for professional continuity. This approach allowed AI-generated responses to be considered alongside the evolving clinical support provided following the mother’s initial concern.

This study is also consistent with the findings of Aslan et al. [23], who reported that AI platforms, particularly ChatGPT) can provide readable, well-structured and medically accurate breastfeeding information. However, the authors also emphasized that AI chatbots may generate plausible but inaccurate or misleading claims, commonly described as ‘’AI hallucinations’’. Similarly, Ball Dunlap et al. [41], emphasized that, although AI may serve as a valuable adjunct for administrative tasks and risk prediction, the human elements of nursing-clinical intuition, compassion, and moral accountability- remain entirely irreplaceable.

The standardized comparison addressed five core areas: milk sufficiency, breast pain, diaper counts, feeding schedules for sleepy infants, and breast refusal. For these standard concerns, the primary guidance from ChatGPT and Gemini showed substantial convergence with the information provided by the Institute of Child Health. Regarding milk supply, both platforms emphasized weight gain, diaper output, and infant behavior as the most reliable indicators of adequacy, while reassuring mothers that softer breasts or low pump volumes are not definitive signs of low production. For inflammatory symptoms, both platforms discussed possible inflammatory breast conditions, including mastitis or blocked ducts, urging clinical evaluation when systemic symptoms were present. Diaper expectations also showed broad convergence across all sources, including the expectation of approximately six or more wet diapers per day after the first several days of life.

Notably, for breast refusal and sleepy newborns, the AI platforms provided more granular practical advice than the corresponding Institute information. This included specific wake-up techniques, such as skin-to-skin contact and undressing, and strategies for managing breast refusal. The breadth of this information illustrates the potential of AI platforms to provide immediate practical guidance, although the present study did not assess whether more detailed responses translated into greater clinical usefulness or improved maternal or infant outcomes. The primary distinctions between platforms were related to response presentation and interaction style: ChatGPT maintained a consistent structure and often sought further clinical context via follow-up questions, whereas Gemini employed more visual formatting, including numbered lists, bolded warnings and images. However, the sheer volume of AI-generated information may pose a challenge for distressed parents, suggesting that the concise nature of professional reference materials may in some circumstances facilitate more immediate identification of key information.

However, convergence with the Institute of Child Health material should be interpreted as a similarity of content rather than independent confirmation of the clinical accuracy of every recommendation. Clinical appropriateness depends on the specific context and on alignment with current evidence and guidelines. This distinction was especially relevant to responses concerning breast inflammation, in which recommendations to “empty” the breast frequently or statements implying that antibiotics are generally required should be interpreted within the current mastitis-spectrum framework [35].

An additional example highlighting the distinction between informational completeness and clinical accuracy emerged in the clinical scenario concerning aversion to breast touch. Gemini used the term “Dysmorphic Milk Ejection Reflex” instead of the established term “Dysphoric Milk Ejection Reflex (D-MER)”. Beyond this terminological error, proposing D-MER as a potential explanation requires careful clinical interpretation because D-MER is characterized by sudden, transient negative emotions temporally associated with the milk ejection reflex and is not necessarily synonymous with generalized aversion to breast touch [36,37,42,43]. This example demonstrates that a detailed and supportive AI-generated response still contains terminological or clinical points requiring professional evaluation.

Clinic-based scenarios revealed critical differences that were less apparent in standardized questions. While AI addressed both informational and emotional aspects, the clinical consultations allowed additional information to emerge through targeted questioning and the interpretation of paralinguistic and contextual cues, including maternal tone, hesitation, fatigue, and home circumstances.

In the scenario of nipple pain, both AI-generated responses and the clinical approach recognized the mother’s concern regarding the continuation of breastfeeding. However, the evolving clinical conversation further highlighted maternal fatigue, the limited support system at home and the emotional distress, allowing the adjustment of the guidance and the arrangement of a follow-up call. In the sensitive scenario of involuntary orgasmic response during breastfeeding, the two AI platforms used reassuring and non-judgmental language, whereas the clinical conversation permitted a deeper exploration of the intensity of guilt and psychological distress and, with the mother’s consent, the arrangement of psychological support. Similarly, the case of a mother having trouble accessing an appropriate breast pump demonstrated an important distinction between recommending a potentially useful resource and facilitating access to that resource. In this case, the clinical service was able to translate the identified need into a practical intervention by facilitating access to a breast pump free of charge, thereby addressing a practical barrier to the mother’s feeding plan.

For sensitive concerns involving breastfeeding-related bodily sensations or tactile aversion, the AI provided detailed biological explanations framed in a reassuring and non-judgmental manner. Yet, the clinic’s approach focused on deep emotional exploration and the provision of psychological support, ensuring that a mother’s mental well-being and personal boundaries remained a priority alongside her nutritional goals for the infant.

Beyond differences in clinical content, the consultations demonstrated the additional contextual and relational information available through direct human interaction. Paralinguistic cues-including vocal volume, pace, hesitation, and crying-could alert the midwife to possible fatigue, anxiety, embarrassment, or a need for privacy and prompt further sensitive questioning. Mothers also frequently referred to distinctive characteristics of their infants, such as “the baby with lots of hair,” to help the midwife remember the family and re-establish an existing relationship with the service. Continuity of contact also allowed the clinical team to draw on previous conversations and, when available and appropriate, relevant clinical information and medical records, as well as to provide subsequent follow-up. Although AI platforms can generate empathetic language, the stand-alone interactions evaluated in this study could not reproduce this relational continuity or independently access clinical records, examine the infant, coordinate with other healthcare professionals, or initiate follow-up.

Within the constraints of text-based communication, ChatGPT and Gemini differed in how they attempted to engage and support the user. ChatGPT maintained a relatively consistent structure across questions and frequently concluded by requesting additional information, such as the infant’s age, weight history, diaper output, or feeding pattern. Gemini generally provided longer and more visibly structured responses, often using numbered instructions, section headings, analogies, warnings, and occasional visual material. Requests for additional information appropriately acknowledged that individualized health guidance depends on clinical context. However, because follow-up interaction was not permitted in the study design, the platforms were not given the opportunity to incorporate such additional information into a subsequent response.

These findings also highlight a potential trade-off between comprehensiveness and usability. Detailed responses may improve completeness by incorporating alternative explanations, management options, and warning signs. Nevertheless, an exhausted, anxious, or distressed mother may find a lengthy response difficult to process, and essential guidance may become less visible among numerous explanations and hypothetical interventions. The value of an AI-generated response therefore depends not only on its accuracy and completeness but also on whether its length, organization, and language enable the mother to identify, understand, and apply the most important information.

The results suggest a potential complementary role for AI in the landscape of breastfeeding support. The findings support the potential usefulness of single-turn AI responses as a direct source of general information for breastfeeding and for identifying situations where professional evaluation is required. However, the clinical scenarios highlighted aspects of care that expanded beyond the single-turn responses of the AI platforms evaluated in this study, particularly when concerns required immediate clinical evaluation, follow-up, coordination with other healthcare professionals, or practical intervention, or when they involved inadequate infant weight gain, potential infection or significant psychological distress.

This study has several limitations. First, the ten breastfeeding-related prompts were purposively selected and may not represent the full range of concerns encountered during pregnancy and the postpartum period. In addition, the two groups of questions had different sources for comparison: responses to the standardized questions were compared with information published by the Institute of Child Health, whereas the clinic-derived scenarios were compared with guidance and management provided by the hospital’s clinical team. The use of the Institute’s of Child Health material as a reference for the standardized questions reflects the available recommendations at the time the study was conducted. However, as scientific evidence and clinical guidelines evolve and are updated, the clinical appropriateness of individual recommendations may change. Therefore, agreement with the reference material should be interpreted primarily as content convergence rather than a timeless confirmation of the clinical accuracy of each individual recommendation. The clinic-derived scenarios originated from a single breastfeeding-support service within a Baby-Friendly Hospital and may therefore reflect the organization, expertise, and continuity of care available in this specific setting. Although the clinic-derived scenarios were based on written records made at the time of the telephone communications rather than subsequent recall by healthcare professionals, documentation bias cannot be excluded, as the records were made in the context of routine clinical practice and may not capture every detail of the initial conversation. Furthermore, the deliberate selection of five clinic-derived scenarios from the available records limits their representativeness and, therefore, does not necessarily capture the full range of communications received by the service. Second, ChatGPT and Gemini responded to isolated written prompts, whereas the clinical team could ask targeted questions, interpret paralinguistic cues, review available medical records and previous contacts, and, where appropriate, arrange follow-up, direct clinical assessment, or practical intervention. Although this design reflected the different ways in which mothers may access AI-based and clinical support, it does not constitute a controlled comparison of clinical competence.

Each question was submitted as an independent, stand-alone prompt without prior conversational history or additional context. No follow-up interaction, prompt reformulation, or regeneration was permitted. This approach was intended to simulate a mother entering a single question into an AI platform and expecting an immediate response. However, it prevented the platforms from using the additional information they requested to refine or individualize their recommendations. Accordingly, the findings should be interpreted as reflecting single-turn AI responses rather than the full capabilities of conversational AI systems during iterative interaction.

Only one response was obtained from each platform for each prompt. Commercial AI outputs may vary according to model updates, proprietary configurations, account characteristics, geographic location, date of use, and other factors that are not transparent to either users or researchers. The findings therefore represent a snapshot of the platform versions available on the two dates of data collection and may not be reproduced identically at a later time. Additionally, the type of prompts was associated with the collection date of the AI responses: the standardized questions were submitted on July 5th, while the clinic-derived scenarios were submitted on July 26 of 2026. Consequently, the differences observed between the two question groups cannot be completely separated from potential changes in platform behavior between the two points. Furthermore, because ChatGPT and Gemini are proprietary systems, differences between their responses cannot be attributed confidently to specific training methods, datasets, or architectural characteristics.

Another limitation is the language of the prompts. All questions were submitted to the AI platforms in English, whereas the clinic derived scenarios originated from telephone communications that had taken place in Greek and were translated into English for the purposes of the study. Although the translation was performed by a pediatrician-neonatologist with an excellent command of the English language, the loss or alteration of subtle linguistic, emotional or cultural tone cannot be ruled out. Moreover, the findings do not necessarily reflect the performance of the same platforms on Greek-language prompts, nor the specific communication needs of a Greek-speaking clinical conversation.

Finally, the responses were evaluated descriptively rather than by blinded independent assessors using validated quantitative instruments, and the study did not measure maternal or infant outcomes.

Future research could expand upon the present single-turn design by comparing structured, multi-turn interactions with AI platforms against corresponding clinical conversations, in which the same information regarding the mother and infant would be provided gradually and in the same sequence. Such an approach would allow the evaluation of both the initial response and the system’s ability to request appropriate clarifications, integrate new clinical and psychosocial data and adjust their guidance accordingly. The development and evaluation of such approaches should also include mothers from diverse educational, linguistic and cultural backgrounds, particularly regarding sensitive issues concerning guilt, physical boundaries or family pressures. At the same time, the evaluation should examine potential, unintended consequences of AI-generated guidance, such as delays in seeking professional care, inappropriate recommendations for complementary feeding, the worsening of psychological distress or over-reliance on reassuring responses. Finally, special attention is required for the protection of privacy and confidentiality, given that mothers may share particularly sensitive information on such platforms regarding lactation, their physical experience and their psychological state. Such studies could also explore hybrid models in which information generated by AI systems is integrated into a framework of professional supervision and clearly defined pathways for accessing personalized clinical care.

## 5. Conclusions

The growing recognition of human milk as more than a source of nutrition, with important implications for infant health and development, has further increased the importance placed on breastfeeding and access to timely and reliable breastfeeding support. At the same time, the increasing integration of Artificial Intelligence into everyday information-seeking is changing the way mothers may seek guidance and reassurance about breastfeeding, highlighting the relevance of understanding both the potential and the limitations of AI-generated support. In the present study, the single-turn responses generated by ChatGPT and Gemini provided relevant, detailed and empathetic information for common breastfeeding inquiries. These findings suggest a potential role for AI-generated responses in providing preliminary breastfeeding information and support, while the clinic-derived scenarios highlighted dimensions of care that extend beyond information provision, including individualized assessment, continuity, direct clinical evaluation, practical intervention, and coordination of care. The increasing availability of AI therefore does not diminish the importance of professional breastfeeding support but reinforces the need to define how these emerging tools can be integrated appropriately alongside human care. True breastfeeding care remains rooted in direct assessment, professional continuity, and the trusted human relationships established within a clinical framework.

## Figures and Tables

**Table 1:** Descriptive comparison of AI-platform responses to standardized breastfeeding questions with corresponding information from the Institute of Child Health (ICH), Greece.

Question	AI Platform	Agreement with ICH	Differences and Additional Information Relative to ICH	Clinical Accuracy and Points Requiring Interpretation	Recommendations for Professional or Clinical Assessment	Attention to Clinical and Contextual Factors
**1. “Am I producing enough milk for my baby?”**	**ChatGPT**	Like the ICH response, recognizes the relationship among infant demand, breastfeeding frequency, and milk production.	Adds observable indicators of adequate intake, including audible swallowing, feeding frequency, wet and soiled diapers, weight gain, and behavior after feeds. It also states that cluster feeding, softer breasts, or a small volume obtained by pumping do not, in isolation, establish insufficient milk production.	Numerical guidance on feeding frequency and diaper counts should be interpreted alongside postnatal age, weight gain, and the infant’s overall clinical condition.	Identifies reduced urine output, difficulty waking, inadequate weight gain, and signs of dehydration as reasons to seek professional assessment.	Requests the infant’s age, wet and soiled diaper counts, and whether the infant is exclusively breastfed or also receives formula, acknowledging the need for individual clinical information.
	**Gemini**	Also recognizes the supply-and-demand relationship described by the ICH and explains that cluster feeding may reflect increased feeding needs and stimulation of milk production.	Adds information on active swallowing, behavior after feeding, feeding frequency, wet and soiled diapers, and weight gain. Also states that soft breasts or a small pumped volume do not necessarily indicate insufficient milk production.	Specific feeding-frequency and diaper-count values are general indicators that require interpretation in relation to the infant’s age, growth, and clinical condition.	Recommends contacting a pediatrician or lactation consultant if the infant is lethargic, has reduced urine output, or raises concerns about weight gain.	Requests the infant’s age and information about wet diapers, recognizing that assessment of intake requires further individual context.
**2. “My breast is red and painful. What should I do?”**	**ChatGPT**	Like the ICH response, identifies a blocked milk duct or mastitis as a possible cause and supports continued breastfeeding.	Adds advice about cold compresses, analgesia, avoiding vigorous massage and excessive pumping, and clinical features that may warrant further assessment.	References to frequent feeding or “emptying” the breast require interpretation considering the current mastitis-spectrum model, which discourages excessive milk removal. The response also advises against excessive pumping and vigorous massage.	Recommends medical assessment for fever or chills, spreading redness, worsening symptoms, a persistent lump, or if symptoms do not improve.	Requests the duration of symptoms, whether fever or chills are present, whether the mother is breastfeeding or pumping, and whether there is a firm or painful area.
	**Gemini**	Includes mastitis and abscess in the differential diagnosis and supports continued breastfeeding or milk expression from the affected breast.	Adds a differential diagnosis, systemic symptoms and warning signs, analgesia, warm or cold compresses, and advice to obtain medical assessment.	The statement that mastitis usually requires antibiotics does not adequately distinguish inflammatory from bacterial mastitis. Advice to “empty” the breast frequently also requires interpretation in light of the mastitis-spectrum model.	Recommends same day medical assessment, with urgent evaluation if high fever or chills, vomiting, spreading redness, an enlarging painful lump, or marked deterioration in general condition occurs.	Differentiates recommendations according to local and systemic symptoms and recognizes the need for clinical examination to determine the cause.
**3. “How many diapers should an infant have each day?”**	**ChatGPT**	Its description of changes in urine and stool output during the first days of life is generally consistent with ICH information.	Extends beyond the first four days by describing expected urine output from day five and changes in stool frequency after the first 4–6 weeks.	For day four, gives a threshold of ≥4 wet diapers, whereas the ICH specifies 6–8 diapers with clear urine. Numerical values should be considered alongside postnatal age, feeding, hydration, and weight gain.	Cites fewer than six wet diapers per day after day five, dark urine or urate crystals beyond the first days, persistently dark stools, difficulty waking, poor feeding, or inadequate weight gain as reasons for assessment.	Adjusts information according to postnatal age and relates diaper output to feeding adequacy and weight gain.
	**Gemini**	Is broadly consistent with the ICH regarding the progressive increase in wet diapers during the first days and describes 6–8 wet diapers after day five.	Groups days 1–2 and 3–4 rather than providing a value for each day. Extends the discussion to older infants and describes changes in stool patterns after the first weeks.	For days 3–4, gives approximately 3–4 wet diapers, whereas the ICH specifies ≥3 on day three and 6–8 on day four. This difference principally concerns the specificity of guidance by day of life.	Identifies fewer wet diapers and dark or concentrated urine as potential signs of inadequate intake or hydration warranting pediatric assessment.	Organizes information by age period, recognizing that expected output changes as the infant grows.
**4. “Should I wake my baby to breastfeed?”**	**ChatGPT**	Like the ICH response, recognizes that the need to wake an infant for feeding depends on age, weight gain, and clinical condition, and that persistent sleepiness warrants assessment.	Distinguishes newborns who have not regained birth weight from older infants gaining weight adequately and specifically addresses preterm and other higher-risk newborns.	Some suggested feeding intervals differ from those in the ICH response. They are general guidance that should be adjusted for gestational age, feeding effectiveness, weight gain, and clinical condition.	Identifies persistent sleepiness, difficulty waking, reduced urine output, inadequate weight gain, fever, hypotonia, respiratory distress, and worsening jaundice as reasons for prompt medical assessment.	Differentiates guidance according to age, weight, recovery of birth weight, prematurity, and the infant’s general condition.
	**Gemini**	Also recognizes that age, weight, and recovery of birth weight affect whether an infant needs to be awakened for feeding.	Adds practical guidance for infants before and after recovery of birth weight and identifies circumstances requiring closer monitoring. Unlike the ICH response, it does not use the same distinction between the first 24 h and the first 10–15 days.	Its emphasis on age and weight must be interpreted alongside feeding effectiveness, gestational age, jaundice, concurrent illness, and urine output.	Recommends pediatric assessment if the infant cannot be awakened or has lethargy, jaundice, or reduced urine output.	Adapts its recommendations to age, weight, and recovery of birth weight and recognizes situations requiring individualized medical guidance.
**5. “My baby refuses to breastfeed. What should I do? “**	**ChatGPT**	Like the ICH response, recognizes that breast refusal may be temporary and identifies possible causes, including illness, nasal congestion, changes in routine or smells, and changes in milk flow.	Suggests skin-to-skin contact, offering the breast when the infant is calm or drowsy, changing breastfeeding positions, and expressing milk to maintain supply. The ICH provides a broader list of possible causes, including maternal factors, oral lesions or infections, artificial nipples, and food allergy.	No clear major clinical inaccuracy was identified in the descriptive comparison. Determining the underlying cause requires an individual history and, when indicated, clinical assessment.	Recommends assessment for reduced urine output, lethargy or poor general condition, signs of dehydration, prolonged breast refusal, or concerns about weight gain.	Recognizes that observation of a complete feed by a lactation consultant may help identify the cause and guide individualized strategies.
	**Gemini**	Also describes breast refusal as generally temporary, referring to a possible “nursing strike,” and identifies factors that may cause infant discomfort, frustration, or distraction.	Suggests practical measures to maintain infant intake and maternal milk production, including expression or pumping and temporary alternative milk feeding. The ICH focuses more on possible causes of refusal. The response also includes visual material related to breastfeeding practices.	The choice of an alternative feeding method and the need for pumping depend on infant age, duration of refusal, adequacy of intake, and clinical condition; these recommendations therefore require individualization.	Emphasizes maintaining adequate intake and seeking professional assessment if refusal persists or there are signs of inadequate feeding or hydration.	Recognizes that breast refusal can have different underlying causes and that management depends on the circumstances contributing to breastfeeding discomfort or difficulty.

**Table 2:** Descriptive comparison of AI-generated responses with the actual clinical management of clinic-derived breastfeeding scenarios.

Original Clinic-Derived Scenario	Source of the Answer	Main Approach/Content	Additional Information and Distinctive Features	Recommendations for Professional/Clinical Assessment/Further Management
**01. “Good evening. My name is M…. I am breastfeeding my baby, but my nipples have become very sore and hurt a lot every time I put my baby to the breast. I’m afraid I won’t be able to continue breastfeeding. What can I do to help them heal and continue breastfeeding without so much pain?”**	**ChatGPT 5.5**	It recognizes both the severe nipple pain and the mother’s fear that she will not be able to continue breastfeeding. It supports continued breastfeeding and focuses on correcting latch and positioning, initiating the feed on the less painful breast, and providing nipple care.	It provides detailed guidance on latch and breastfeeding positions, the use of expressed breast milk and air-drying, lanolin/hydrogel dressings, avoidance of irritating cleansers and pain relief. It also mentions possible causes of persistent pain, such as suboptimal latch and infectious causes.	It recommends assessment by a lactation professional/healthcare provider when the pain is severe or persistent, there are cracks or bleeding, inadequate weight gain/urination, or signs of infection.
	**Gemini 3.5 Flash**	It also acknowledges the mother’s pain and emotional distress and supports the possibility of continuing breastfeeding. It places particular emphasis on optimizing latch and positioning.	It provides detailed instructions on achieving a deep latch, changing positions, using breast milk, lanolin/nipple balm, silver nursing cups and hydrogel pads and mentions tongue-tie and thrush among the possible causes. The response also includes visual material illustrating a shallow versus deep latch, complementing the written practical guidance.	It recommends a hands-on assessment by an IBCLC or another healthcare professional when the pain persists despite correcting the latch, or when there are deep cracks, bleeding or shooting pain.
	**Clinical management**	The clinical conversation was not limited to addressing the pain; it also explored feeding practices, maternal fatigue and the broader psychosocial context. It was established that the mother was essentially taking care of her 8-day-old newborn alone during the day, as her husband had returned to work.	Through targeted questions, it was noted that she sometimes waited until the infant cried before breastfeeding and that she allowed the infant to remain latched after falling asleep. Rest, hydration and self-care were also explored. When the mother became tearful and expressed fear that she would fail infant care, emotional support and reassurance were provided.	An in-person assessment of the mother and the infant was offered, and follow-up was arranged for the following day.
**02. “Our pediatrician told us that the baby is not gaining enough weight and recommended that I start giving formula. However, I really want to continue exclusive breastfeeding. I don’t know what to do, and I’m afraid of making the wrong decision. How should I proceed?”**	**ChatGPT 5.5**	It recognizes the dilemma between the desire to maintain exclusive breastfeeding and the need to ensure adequate infant growth. It recommends objectively confirming weight gain and assessing the effectiveness of breastfeeding.	It provides information on assessing milk intake/transfer through weight trajectory, diaper output, latch, swallowing and weighted feed. It recommends more frequent breastfeeding, offering both breasts and using breast compressions. If supplementation is required, it recommends pumping to maintain milk supply and using expressed breast milk when available. It does not recommend overriding the pediatrician’s advice and emphasizes that, if inadequate nutrition is present, necessary intervention should not be delayed.	It recommends an IBCLC assessment, weight monitoring and collaboration with the pediatrician.
	**Gemini 3.5 Flash**	It also acknowledges the mother’s confusion and presents adequate infant growth and continuation of breastfeeding as goals that can coexist. It recommends an objective assessment of milk transfer and the infant’s growth.	It recommends an IBCLC assessment, a weighted feed, evaluation of latch, oral anatomy and feeding efficiency. If the pediatrician considers supplementation necessary, it presents it as a temporary “bridge’’, suggesting expressed breast milk, SNS, paced bottle/cup feeding and pumping to protect milk supply. It also recommends setting a specific weight-gain target and reassessing in 3–7 days.	It recommends an immediate IBCLC assessment, weight monitoring and collaboration with the pediatrician.
	**Clinical management**	The clinical team explored the mother’s concerns and proceeded with an immediate objective assessment of the infant, rather than relying solely on the reported inadequate weight gain.	The mother and the infant presented to the hospital, where a clinical examination and weight assessment were performed. The infant was clinically well and adequate weight gain with exclusive breastfeeding was confirmed. The team subsequently contacted the infant’s pediatrician directly.	A shared follow-up plan was agreed upon with the pediatrician so that the mother would receive consistent and coordinated guidance.
**03. “Please, I need your help. My baby is 10 days old, and sometimes when I breastfeed, I experience an orgasm. I feel extremely embarrassed and overwhelmed with guilt. I’m thinking about stopping breastfeeding, but my husband and my mother-in-law insist that I continue, and I can’t tell them what is happening to me. Is this normal? Has this happened to other women as well? What should I do?”**	**ChatGPT 5.5**	It acknowledges both the physical phenomenon and the intense shame and guilt described by the mother. It reassures her that this is an involuntary bodily response that has also been reported by other breastfeeding women and does not indicate inappropriate intent or feelings toward the infant.	It provides a physiological explanation, referring to oxytocin and the let-down reflex, along with practical strategies for managing the discomfort. It also acknowledges the family pressure and emphasizes that the mother is not obliged to disclose this personal experience to relatives.	It recommends confidential support from a lactation professional, midwife/maternity care professional or mental health professional when the experience causes significant psychological distress.
	**Gemini 3.5 Flash**	It also recognizes the response as involuntary, provides emotional validation and clearly distinguishes it from sexual intent toward the infant. It also acknowledges the mother’s need for privacy.	It provides a more detailed physiological explanation, referring to oxytocin, nipple stimulation, uterine/pelvic contractions and postpartum physiological changes and suggests practical changes in positioning, environment and distraction.	It recommends support from an IBCLC or a professional with experience in perinatal mental health when the experience remains significantly distressing.
	**Clinical management**	The clinical approach provided nonjudgmental information and reassurance while also further exploring the frequency of the episodes, the breastfeeding pattern and most importantly, the intensity of the guilt, shame and psychological distress.	The discussion allowed for an individualized assessment of the emotional burden and family context, beyond the physiological interpretation of the symptom.	With the mother’s consent, an actual appointment for psychological support was arranged, rather than simply providing a general recommendation to seek help.
**04. “My baby is unable to latch onto my nipple, and so far I have been expressing my milk by hand to feed the baby. However, I am completely exhausted and am thinking about stopping breastfeeding. How can I wean safely? Is there any other solution?”**	**ChatGPT 5.5**	It acknowledges the exhaustion caused by continuous hand expression and does not treat weaning as the only option. It recommends investigating the underlying cause of the latching difficulty and exploring alternative ways to maintain the mother’s milk supply.	It recommends skin-to-skin contact, a lactation assessment, use of a breast pump and in selected cases, a nipple shield/SNS. It also provides guidance on gradual and safe weaning if that is the mother’s choice.	It recommends professional assessment of the latch and medical evaluation for symptoms consistent with mastitis during the weaning process.
	**Gemini 3.5 Flash**	It also acknowledges the physical and psychological burden of continuous hand expression of the breast milk and presents options both for continuing to provide breast milk and for safe, gradual weaning.	It recommends an IBCLC, a nipple shield, a double electric breast pump, exclusive pumping, combination feeding and detailed measures for gradual weaning and managing breast fullness.	It recommends an IBCLC assessment and medical evaluation if signs of mastitis develop.
	**Clinical management**	The evolving clinical conversation revealed that the main barrier in using a breast pump was not the mother’s reluctance, but her inability to afford one.	The clinical team went beyond providing advice: they contacted a partner company and secured a free breast pump for the mother.	At follow-up, it was confirmed that the mother was using the breast pump successfully and continued to provide breast milk to the infant.
**05. “I hate having someone touch my breasts, but I support breastfeeding. I would like to breastfeed my baby, but I can’t stand the sensation. What can I do?”**	**ChatGPT 5.5**	It recognizes that the desire to breastfeed can coexist with a strong aversion to physical contact and addresses the discomfort without judgment. It explores possible sensory, physical and emotional dimensions.	It recommends exploring the triggers, optimizing positioning/latch, reducing sensory overload, pumping or hand expression and considering alternative ways of providing breast milk.	It recommends individualized support from a lactation professional and, when indicated, from another healthcare or mental health professional.
	**Gemini 3.5 Flash**	It also acknowledges the sensory and psychological dimensions of the aversion and presents options that can reduce unwanted direct breast touch while maintaining, if desired, the ability to provide breast milk.	It recommends a nipple shield, hands-free/exclusive pumping, sensory distraction and combination feeding, and mentions D-MER as a potentially related phenomenon. The response also includes visual material on how to apply a silicone nipple shield, supplementing the written practical instructions.	It recommends individualized support from an IBCLC, including trauma-informed/sensory-informed lactation care.
	**Clinical management**	The clinical approach avoided initially attributing the aversion to a specific cause and used targeted questions to clarify its nature and triggers.	It was explored whether the discomfort occurred only during direct breastfeeding or also during hand expression of milk or pumping, as well as whether it was associated with pain, anxiety, previous negative experiences, or other unpleasant sensations.	Further guidance could be tailored to the information that emerged during the ongoing clinical conversation and to the mother’s preferences.

## Data Availability

All data supporting the findings of this study are included within the article.

## Supplementary Materials

The following supporting information can be downloaded at https://www.mdpi.com/article/10.3390/jcm15197513/s1, Table S1: Frequently Asked Questions of Institute of Child Health. Table S2: Comparison of AI-generated responses with real-world clinical support for clinic-derived breastfeeding scenarios.
