# Supplementary material for: In the Age of Artificial Intelligence: From Standardized Questions to Real-World Breastfeeding Concerns
Source: J Clin Med. 2026 Sep 27;15(19):7513. doi: 10.3390/jcm15197513 (PMC13665832; doi:10.3390/jcm15197513)
Supplement: Supplementary file 1 [file jcm-15-07513-s001.zip › jcm-4584156-supplementary.pdf]

Table S1. Frequently Asked Questions of Institute of Child Health.

Table 1. Frequently Asked Questions of Institute of Child Health	
01. "Do I have enough milk for my baby?"	
**Institute of Child Health-Greece**	**Breast milk production adjusts according to the baby's needs. The amount of milk a mother can produce depends on the baby's age and adapts to the infant's changing requirements. As the baby grows, the capacity of the stomach increases, and milk requirements also increase. During certain periods, this may lead to more frequent breastfeeding, which helps stimulate the breast to produce more milk.**
**ChatGPT 5.5** ***(on 05-07-2026)***	**In most cases, the best way to tell whether your baby is getting enough milk is how they're growing and behaving, not how your breasts feel or how much milk you can pump.**<br>**Signs your baby is getting enough milk include:**<br>**• At least 6 wet diapers per day after about 5 days of age.**<br>**• Regular bowel movements (the pattern varies as babies get older, especially after the first month).**<br>**• They seem satisfied and relaxed after most feeds.**<br>**• You can hear or see them swallowing while feeding.**<br>**• They are gaining weight appropriately, as confirmed by their healthcare provider.**<br>**• They are alert and meeting expected developmental milestones.**<br>**Things that do not necessarily mean you have a low milk supply:**<br>**• Your breasts feel softer than they did in the first few weeks.**<br>**• Your baby wants to feed frequently (cluster feeding is common).**<br>**• You pump only a small amount of milk (babies are often much more effective than pumps).**<br>**• Your baby wants to nurse for comfort as well as nutrition.**<br>**When to seek help promptly**<br>**Contact your baby's healthcare provider or a lactation consultant if your baby:**<br>**• Has fewer than 6 wet diapers a day after day 5.**<br>**• Is very sleepy and difficult to wake for feeds.**<br>**• Is not gaining weight or is losing weight after the first few days.**<br>**• Shows signs of dehydration, such as a very dry mouth, dark urine, or no tears when crying (in older infants).**<br>**If you tell me:**<br>**1. How old is your baby? (days or weeks old)**<br>**2. How many wet and dirty diapers they have in 24 hours?**<br>**3. Whether they are exclusively breastfed or also receive formula,**<br>**I can help you assess whether your milk intake sounds adequate.**
**Gemini 3.5 Flash** ***(on 05-07-2026)***	**Provided for informational purposes only. For medical advice or diagnosis, consult a healthcare professional. This is one of the most common worries for new parents, and it can feel incredibly stressful**

	when you can't physically see how many ounces your baby is taking in. Because your body operates on a supply-and-demand system, your mind can easily trick you into thinking you're running low-especially during growth spurts.<br>The most reliable way to know your baby is getting enough milk isn't by looking at your breasts or a pump dial; it's by looking at what goes *into* the diaper and how your baby is growing.<br>1. The "Input" Signs (Behavior)<br>● Active swallowing: You can hear or see a rhythmic swallow during a feed (a "deep jaw drop" pause).<br>● The "Milk Drunk" state: Your baby relaxes their hands, drops their shoulders, and often falls asleep contentedly after a feeding.<br>● Feed frequency: Newborns typically feed 8 to 12 times in a 24-hour period.<br>2. The "Output" Signs (Diapers)<br>What goes in must come out. After the first week of life, a well-hydrated baby should consistently produce:<br>● Wet diapers: 6 or more heavily wet diapers every 24 hours. The urine should be pale and odorless, not dark or concentrated.<br>● Poop diapers: Usually 3 or more yellow, seedy stools a day (though after 4–6 weeks, some healthy breastfed babies poop less frequently, even going a few days between stools).<br>3. The Ultimate Tracker: Weight Gain<br>Your pediatrician's scale is your best source of truth. After dropping a bit of weight right after birth, a healthy baby should regain their birth weight by 10 to 14 days old and continue gaining along their personal growth curve.<br>Common False Alarms:<br>● Soft breasts: Breasts often feel softer after the first few weeks. This doesn't mean your milk disappeared; it just means your body has regulated its supply to match exactly what your baby needs.<br>● Cluster feeding: If your baby wants to nurse constantly for a few hours, it's usually a normal growth spurt, not a sign of low supply. They are "ordering" more milk for tomorrow.<br>● Low pump output: A pump is never as efficient as a baby. Pumping only a small amount does *not* mean your baby isn't getting plenty when they nurse.<br>If your baby seems constantly lethargic, isn't wetting diapers, or you are worried about weight gain, always trust your gut and give your pediatrician or a lactation consultant a quick call. How old is your baby right now, and how are those wet diapers looking?
02. “My breast is red and painful. What should I do?”	
Institute of Child Health-Greece	Redness of the breast is most commonly due to a blocked milk duct or mastitis. Effective emptying of the breast is the first-line approach in both cases. Positioning the baby so that the chin or nose points toward the affected area may help relieve the blockage more effectively. If the redness spreads or persists, consultation with a healthcare professional is necessary.
ChatGPT 5.5 *(on 05-07-2026)*	A red, painful breast during breastfeeding can have several causes. The most common are a plugged (blocked) milk duct or mastitis

	(inflammation of the breast, which may or may not involve infection).<br>What you can do right away<br>● Continue breastfeeding or expressing milk from the affected breast. Emptying the breast usually helps, and it's safe for your baby to keep feeding.<br>● Feed frequently, starting on the affected side if it's not too painful.<br>● Use cold packs (wrapped in a cloth) for 10–20 minutes after feeds to reduce pain and swelling.<br>● Take pain relief if needed, such as Ibuprofen or Paracetamol, provided they're appropriate for you. Both are generally considered compatible with breastfeeding.<br>● Wear a supportive, non-tight bra and avoid pressure on the affected area (tight clothing, underwire bras, or sleeping on that side).<br>Avoid<br>● Aggressive breast massage or trying to "push out" a blockage, as this can make inflammation worse.<br>● Excessive pumping beyond what your baby normally needs, as this can increase milk production and worsen the problem.<br>Seek medical attention promptly if:<br>● You have a fever (38.5°C or higher), chills, or flu-like symptoms.<br>● The redness is spreading or the pain is getting worse.<br>● You notice a painful lump that doesn't improve.<br>● Symptoms don't start improving within 12–24 hours despite frequent feeding and supportive care.<br>● You see pus or have persistent nipple discharge other than milk.<br>If mastitis is suspected, you may need antibiotics, and starting treatment early can help prevent complications such as a breast abscess.<br>If you can tell me:<br>● How long your breast has been red and painful,<br>● Whether you have a fever or chills,<br>● Whether you're breastfeeding, pumping, or both,<br>● And whether you feel a hard lump,<br>I can help you narrow down the most likely cause and advise on the next steps.
Gemini 3.5 Flash *(on 05-07-2026)*	Provided for informational purposes only. For medical advice or diagnosis, consult a healthcare professional. I'm really sorry you are dealing with this. A red and painful breast can be incredibly uncomfortable, and it's something you should take seriously. Because redness, pain, and swelling in the breast can point to a few different issues-some of which require prescription treatment-the most important step you can take right now is to contact a healthcare provider.<br>Here is what you should do right away, along with how to manage the discomfort:<br>1. Call Your Doctor or Visit Urgent Care

	You need a professional evaluation to determine exactly what is causing the inflammation. It could be:<br>● Mastitis: An infection of the breast tissue, which is especially common if you are currently breastfeeding or chestfeeding, but can happen to anyone. Mastitis usually requires a course of prescription antibiotics to clear up.<br>● A Breast Abscess: A localized collection of pus that sometimes develops if a breast infection isn't treated early.<br>● Other Inflammatory Conditions: Less commonly, certain types of severe inflammation or other breast conditions can cause these symptoms and need to be ruled out by a doctor.<br>2. Check for "Red Flag" Symptoms<br>Go to urgent care or the emergency room if your symptoms are severe or accompanied by any of the following:<br>● A high fever or chills<br>● Nausea or vomiting<br>● Red streaks spreading from the painful area across your breast<br>● A hard, very tender lump that feels like it's growing<br>● Feeling suddenly dizzy, confused, or generally very unwell (signs that an infection might be spreading)<br>3. Managing the Comfort (While You Wait to See a Doctor)<br>While you are waiting for your medical appointment, you can use these self-care measures to help ease the pain:<br>● Apply Warm or Cold Compresses: A warm, damp washcloth or a warm shower can help soothe the pain and promote blood flow. Alternatively, a cold pack wrapped in a cloth can help reduce swelling. Use whichever feels best to you.<br>● Take Over-the-Counter Pain Relief: Acetaminophen or ibuprofen can help manage both the pain and the inflammation, provided you have no medical reasons to avoid them.<br>● Wear a Supportive, Non-Wire Bra: Avoid tight clothing or underwire bras that compress the breast tissue, as this can worsen the irritation.<br>● If You Are Breastfeeding: Do *not* stop nursing or pumping on the affected side. Emptying the breast frequently is actually one of the best ways to keep milk from pooling and worsening an infection. Start feedings on the unaffected side first to trigger your milk let-down, then move to the painful side.<br>Please don't wait to see if it just goes away on its own. Reaching out to a doctor today will help ensure you get the right treatment and start feeling better quickly.
03. "How many diapers should a baby have per day?"	
Institute of Child Health-Greece	Day 1: One or more wet diapers; 1-2 stools with meconium<br>Day 2: Two or more wet diapers; 1-2 stools with meconium or transitional stools<br>Day 3: Three or more wet diapers; Transitional stools<br>Day 4: 6-8 diapers with clear urine; Soft yellow seedy stools, at least one diaper per day
ChatGPT 5.5	The number of wet and dirty diapers is one of the best ways to tell if a baby is getting enough milk.

(on 05-07-2026)

Here are general guidelines for healthy, full-term newborns:

Baby's age	Wet diapers	Dirty diapers
Day 1	At least 1	1 or more (dark meconium)
Day 2	At least 2	1–2
Day 3	At least 3	2 or more (becoming greener)
Day 4	At least 4	3 or more (transitioning to yellow[illegible]
Day 5 and onward	At least 6 heavy wet diapers per day	At least 3–4 yellow, loose stools per [illegible] during the first several weeks (som[illegible] babies stool more often)

After about 4–6 weeks, it's normal for breastfed babies to stool less frequently. Some may have one bowel movement every few days, as long as:

- The stools are soft (not hard or pellet-like).
- The baby is feeding well.
- Weight gain is appropriate.
- The baby seems otherwise healthy.

When to seek advice

Contact your baby's healthcare provider or a lactation consultant if:

- Your baby has fewer than 6 wet diapers per day after day 5.
- Urine is dark or has orange "brick dust" crystals after the first few days.
- Stools remain dark (black/green) after day 4–5 instead of turning yellow.
- Your baby is difficult to wake for feeds, isn't feeding well, or isn't gaining weight.

Keeping track of diaper counts during the first week or two can be very helpful in monitoring whether breastfeeding is going well.

Gemini 3.5 Flash *(on 05-07-2026)*

The number of diapers a baby goes through depends entirely on their age. Newborns require the most changes, and the number gradually decreases as their bladder capacity grows.

Here is what to expect by stage:

Newborns (First Week)

In the first few days, the number builds up as the baby takes in more milk:

- Days 1–2: About 1 to 2 wet diapers and 1 to 2 dark, sticky stools (meconium) per day.
- Days 3–4: Around 3 to 4 wet diapers and lighter, transitional stools.
- Day 5 and beyond: The baseline stabilizes. You should expect 6 to 8 wet diapers and at least 3 to 4 dirty diapers daily.

Infants (1 to 6 Months)

Once a baby is a few weeks old, their routine settles into a steady rhythm:

- Wet Diapers: 6 to 8 per day. Wet diapers should feel heavy.
- Dirty Diapers: This varies wildly based on diet. Breastfed babies might poop after every feeding (6+ times a day) or go several

	days without one. Formula-fed babies usually poop 1 to 2 times a day. Both patterns are completely normal.<br>Older Babies (6 to 12+ Months)<br>As babies start solid foods and their bladders grow, the frequency drops slightly:<br>● Total Diapers: Around 5 to 7 per day.<br>When to call the pediatrician: If your baby is over 5 days old and has fewer than 6 wet diapers in a 24-hour period, or if you notice dark, concentrated urine, it can be a sign of dehydration.
04. "Do I need to wake my baby up to feed? / My baby sleeps and does not feed. What should I do?"	
Institute of Child Health-Greece	A healthy newborn does not usually need to be awakened for feeding, unless the pediatrician or healthcare professional recommends otherwise. For example, prolonged jaundice, excessive neonatal weight loss, or reduced wet or dirty diapers may require a more intensive feeding pattern.<br><br>My baby sleeps and does not feed. What should I do?<br>During the first 24 hours, the baby may sleep for many hours and breastfeed only a few times. After the first 24 hours, if the baby sleeps a lot, it is important to wake the baby every 2-3 hours during the day, counting from the beginning of the previous feed, and at least every 5 hours during the night for breastfeeding. This is important during the first 10-15 days in order to stimulate and ensure adequate milk production.<br><br>If a baby sleeps for longer periods and does not respond to attempts by the parents to wake them, this may raise concern for jaundice, fever, or another condition. Immediate communication with the pediatrician is necessary.
ChatGPT 5.5 *(on 05-07-2026)*	It depends on your baby's age, weight, and overall health.<br>For newborns (especially during the first 2–3 weeks):<br>● Yes, you should usually wake your baby to feed if they sleep longer than:<br>o 3 hours during the day, or<br>o 4 hours at night.<br>● Frequent feeding (at least 8–12 times in 24 hours) helps ensure your baby gets enough milk, supports healthy weight gain, and helps establish your milk supply.<br>Once your baby:<br>● has regained their birth weight,<br>● is gaining weight well,<br>● has plenty of wet and dirty diapers, and<br>● your healthcare provider is satisfied with their growth,<br>you can usually let your baby wake naturally for feeds. Some babies will begin sleeping for longer stretches at night.<br>If your baby is very sleepy and difficult to wake:<br>Try to:<br>● Undress your baby down to the diaper.<br>● Gently rub their back, feet, or hands.

	● Change the diaper before feeding.<br>● Hold your baby skin-to-skin.<br>● Offer the breast as soon as they show early feeding cues (stirring, rooting, sucking on hands) rather than waiting until they cry.<br>When to seek medical advice<br>Contact your baby's healthcare provider promptly if your baby:<br>● Is consistently too sleepy to feed or refuses several feeds.<br>● Has fewer wet diapers than expected.<br>● Is difficult to wake.<br>● Is not gaining weight appropriately.<br>● Shows signs of illness such as fever, poor muscle tone, breathing difficulties, or jaundice that seems to be worsening.<br>If your baby is younger than a few weeks old or was born prematurely, always follow the feeding schedule recommended by your pediatrician, as these babies often need to be woken regularly for feeds.
Gemini 3.5 Flash *(on 05-07-2026)*	Provided for informational purposes only. For medical advice or diagnosis, consult a healthcare professional. Whether you need to wake your baby to feed depends almost entirely on one main factor: their age and weight.<br>Here is the general rule of thumb followed by pediatricians:<br>The Golden Rule: Look at the Weight<br>● If your baby is a newborn (under 2 weeks) OR hasn't regained their birth weight: Yes, you should wake them. Newborns have tiny stomachs and can drop their blood sugar quickly if they sleep too long. The American Academy of Pediatrics (AAP) recommends waking them if it has been 3 to 4 hours since their last feed (counting from the start of the last feed). Expect about 8 to 12 feedings in a 24-hour period.<br>● If your baby has regained their birth weight and is growing steadily: No, let them sleep! Once your pediatrician confirms they are gaining weight well and hitting their milestones, you can celebrate and let them sleep until they wake up naturally.<br>What to Do If Your Baby is Too Sleepy to Feed<br>Newborns can be notoriously deep sleepers, sometimes because they are still recovering from birth. If it is time to feed but they won't wake up or keep nodding off at the breast or bottle, try these gentle tricks:<br>● Change their diaper first: The movement and cooler air are usually enough to get them alert.<br>● Strip them down: Go down to just a diaper. Skin-to-skin contact on your chest is an amazing natural trigger that wakes babies up and stimulates their feeding instincts.<br>● Keep it dim but active: Don't turn on bright lights (which will make them squeeze their eyes shut), but do talk to them and gently stroke their hands or feet.<br>● Switch sides or compress: If they drift off mid-feed, switch sides or use breast compression to increase the milk flow, which usually alerts them.

	⚠ When to Call the Pediatrician<br>If your baby is consistently impossible to rouse for multiple feeds in a row, seems completely lethargic (weak, limp, or won't cry), shows yellowing of the skin or eyes (jaundice), or is producing fewer than 4 to 6 wet diapers a day, skip the wake-up tricks and call your doctor immediately.
	05. "My baby refuses the breast. What should I do?"
Institute of Child Health-Greece	Breast refusal, also called a "nursing strike," is a normal phenomenon that may occur in any baby, but it can be particularly discouraging for the mother. She may worry that the baby is rejecting her and her breast.<br><br>Many factors may contribute to breast refusal, including:<br>1. Difficult events during labor or immediately after birth.<br>2. Developmental stage: as babies become more aware of their environment, they may be more easily distracted during breastfeeding. A quiet breastfeeding environment is recommended.<br>3. Negative oral experiences, such as tooth pain, fungal infection, oral ulcers, or herpes simplex type 1 infection.<br>4. Maternal health problems, such as mastitis, pain, malaise, anxiety, or fever.<br>5. Infant health problems, such as otitis media, nasal congestion from a cold, gastroesophageal reflux causing pain during feeding in a horizontal position, or reduced appetite due to temporary illness.<br>6. Use of artificial nipples.<br>7. Milk supply issues, such as low milk production causing frustration, or a strong milk flow causing the baby to gag or vomit.<br>8. Changes in the taste of breast milk due to mastitis, menstruation, pregnancy, medications, deodorants, or perfumes applied near the breast.<br>9. Food allergy.<br>10. Fear related to a strong milk flow or the mother's reaction to nipple pain. Occasionally, a baby may bite while pulling away from the breast. The mother's pain response may frighten the baby.
ChatGPT 5.5 *(on 05-07-2026)*	It can be worrying when your baby refuses the breast, but in many cases it is temporary and can be resolved with patience and support.<br>Common reasons include:<br>• Your baby is not very hungry or is too hungry and upset to latch.<br>• Sleepiness, illness, nasal congestion, or teething.<br>• Changes in routine, unfamiliar smells (such as perfume), or stress.<br>• Difficulty with positioning or latch.<br>• A fast or slow milk flow that frustrates the baby.<br>What you can do:<br>• Stay calm and avoid forcing your baby to the breast.<br>• Offer the breast when your baby is calm, sleepy, or just waking up.<br>• Have plenty of skin-to-skin contact to encourage natural feeding cues.

	• Try different breastfeeding positions.<br>• Hand express a little milk before feeding so your baby immediately tastes milk when latching.<br>• Feed in a quiet, dimly lit environment with minimal distractions.<br>• If your baby is too upset to latch, comfort them first, then try again.<br>If your baby continues to refuse the breast:<br>• Express milk regularly (by hand or with a pump) to protect your milk supply.<br>• Feed your expressed milk using the method recommended by your healthcare provider or lactation consultant, if needed.<br>Seek prompt medical advice if:<br>• Your baby has fewer wet diapers than expected.<br>• Your baby is very sleepy, difficult to wake, or appears unwell.<br>• Your baby shows signs of dehydration (dry mouth, no tears when crying, sunken soft spot, or significantly fewer wet diapers).<br>• Breast refusal lasts more than 24 hours in a young infant or is accompanied by poor feeding or weight concerns.<br>A lactation consultant can observe a full feeding, identify the underlying cause, and provide personalized strategies to help your baby return to breastfeeding.
Gemini 3.5 Flash *(on 05-07-2026)*	Provided for informational purposes only. For medical advice or diagnosis, consult a healthcare professional. It can be incredibly stressful and emotional when your baby suddenly refuses to nurse. This is often called a nursing strike, and it is almost always temporary.<br>Your most important immediate task is to keep feeding your baby using an alternative method (like a bottle, cup, or spoon) and to pump or express milk to protect your milk supply while you sort this out.<br>Why Is This Happening?<br>Babies usually refuse the breast because something is making the experience uncomfortable, frustrating, or distracting for them.<br>Common culprits include:<br>• Physical discomfort: Teething pain, an ear infection, a cold making it hard to breathe through their nose, or soreness from a recent vaccination.<br>• Flow issues: Your milk flow might be too fast (causing them to choke) or too slow (causing them to get frustrated).<br>• Changes or distractions: A new soap or deodorant, a stressful environment, or a sudden loud noise that scared them the last time they nursed.<br>Immediate Steps to Try<br>1. Change the Environment and Position<br>Try nursing in a dark, quiet room with minimal distractions.<br>Sometimes changing how you hold your baby can relieve physical pressure on a sore ear or body part.

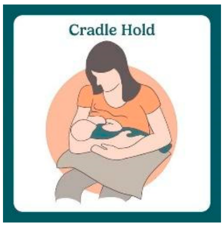

Standard Cradle Hold. Source: The Lactation Collection / cradle hold - Breastfeeding Glossary by The Lactation Collection

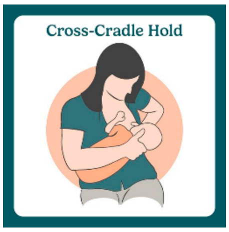

Cross-Cradle Hold. Source: The Lactation Collection / cross-cradle hold - Breastfeeding Glossary by The Lactation Collection

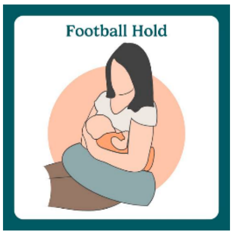

Football Hold. Source: The Lactation Collection / football hold - Breastfeeding Glossary by The Lactation Collection

2. Focus on Skin-to-Skin Contact

Spend time holding your baby against your bare chest with just their diaper on. Do this without any pressure to nurse. This releases calming hormones for both of you and triggers natural biological feeding instincts.

3. Catch Them While Drowsy

Many babies will happily nurse when they are half-asleep or just waking up because their defenses are down and their sucking reflex takes over automatically.

4. Adjust the Milk Flow

- If flow is too slow: Pump or massage your breast for a minute before offering it so the milk is already flowing when they latch.
- If flow is too fast: Express a little milk beforehand to take the edge off the initial spray, or try nursing while leaning backward so gravity slows the milk down.

⚠ When to Call the Pediatrician

While a nursing strike is usually just a phase, you should contact your doctor right away if your baby shows signs of dehydration (fewer wet diapers than normal, no tears when crying, or extreme lethargy) or if you suspect they are in pain from an illness.

If the strike lasts more than a couple of days, reaching out to a certified lactation consultant (IBCLC) can give you personalized, hands-on support to get back on track.

Table S2. Comparison of AI-generated responses with real-world clinical support for clinic-derived breastfeeding scenarios.

01. "Good evening. My name is M…. I am breastfeeding my baby, but my nipples have become very sore and hurt a lot every time I put my baby to the breast. I'm afraid I won't be able to continue breastfeeding. What can I do to help them heal and continue breastfeeding without so much pain?"	
**Clinic Response**	The initial conversation revealed that the mother's husband had recently returned to work and that she was caring for the eight-day-old infant alone during the day. The mother reported that the baby was gaining weight and producing frequent wet and soiled diapers, but that her nipple pain had intensified since discharge. Feeding frequency and duration were explored to identify breastfeeding practices that might be contributing to the pain. The discussion revealed that the infant was sometimes offered the breast only after beginning to cry and was allowed to remain attached after falling asleep.<br>The mother was advised to recognize and respond to early hunger cues so that the infant could feed more calmly. If the baby fell asleep before completing a feed, gentle stimulation was recommended; if the feed had ended, she was advised to release the latch carefully rather than leave the infant sleeping at the breast. Correct positioning and attachment were reviewed. She was also advised to apply lanolin cream or normal saline after, rather than immediately before, breastfeeding; apply a small amount of expressed breast milk to the nipple; avoid frequent washing with soap; and allow the nipples to remain exposed to the air for periods during the day. If the infant was particularly hungry or unsettled, she could begin feeding from the less painful breast.<br>Attention was also given to the mother's wider well-being, including whether she was resting adequately, maintaining hydration, and caring for herself while managing the baby largely alone. When she became tearful and expressed fear that she would not succeed, the midwife normalized the emotional difficulties commonly experienced during the early postpartum period, acknowledged what the mother had already achieved, and reassured her that nipple trauma is common and usually improves with appropriate support.<br>The mother was encouraged to contact the clinic again with any further concerns. An in-person assessment at the maternity hospital was offered if attachment difficulties persisted, and, with her agreement, follow-up communication was arranged for the following day. Further contact could be repeated as needed to monitor her progress and provide continued support.
**ChatGPT 5.5** *(on 26-07-2026)*	I'm sorry you're going through this, M.. Sore nipples are one of the most common reasons mothers think about stopping breastfeeding, but in many cases they can heal while you continue breastfeeding. The most important step is to identify and correct the cause of the soreness. Here are some things that can help:<br>• **Check your baby's latch.** The most common cause of nipple pain is a shallow latch. Your baby should take a large portion of the areola

into their mouth-not just the nipple. Their lips should be flanged outward, and you should hear swallowing rather than clicking sounds.

- **Try different breastfeeding positions.** Positions such as the cross-cradle, football hold, or side-lying position may help your baby latch more deeply and reduce pressure on sore areas.
- **Start feeding on the less painful side first.** Once your milk has let down, switch to the sore side, as your baby may suck more gently.
- **Express a few drops of breast milk after each feed** and gently spread it over the nipple. Allow the nipples to air dry. Breast milk has natural healing properties.
- **Use purified lanolin or a hydrogel dressing** if recommended by your healthcare provider or lactation consultant. These products can help soothe and protect damaged nipples.
- **Keep nipples dry between feeds** and change breast pads frequently if they become wet.
- **Avoid harsh soaps or alcohol-based products** on your nipples, as these can increase dryness and irritation.
- **Take pain relief if needed.** Medications such as ibuprofen or acetaminophen are generally considered compatible with breastfeeding when used as directed, but check with your healthcare provider if you have any medical conditions or concerns.

When should you seek help?

It's important to contact a lactation consultant, midwife, or healthcare provider if:

- The pain is severe or continues throughout the entire feed.
- Your nipples are cracked, bleeding, or not improving after a few days.
- Your baby is having trouble gaining weight or producing fewer wet diapers.
- You notice signs of infection, such as increasing redness, warmth, swelling, pus, or fever.
- You have sharp, burning pain during or after feeds, especially if your baby has white patches in their mouth, as this may suggest a yeast infection.

Can you continue breastfeeding?

In most cases, **yes**. Continuing to breastfeed while addressing the cause of the pain is usually the best approach. Stopping breastfeeding suddenly can lead to engorgement and increase the risk of blocked ducts or mastitis.

If you're comfortable answering, I have a few questions that can help narrow down the cause:

1. Are your nipples **cracked, bleeding, or just very sore**?
2. Does the pain **improve after your baby starts feeding, or does it last throughout the entire feed**?
3. How old is your baby?
4. Have you noticed **clicking sounds**, slipping off the breast, or a white coating in your baby's mouth?

**Gemini 3.5 Flash** *(on 26-07-2026)*	Hi M.. Please take a deep breath - you are doing a wonderful job, and it is completely understandable to feel overwhelmed when feeding causes this much pain.

Sore or cracked nipples are extremely common in the early weeks, but **breastfeeding is not supposed to hurt continuously**. Pain is almost always a sign of a **shallow latch**, where the nipple gets compressed against the hard roof of the baby's mouth rather than resting comfortably in the soft palate at the back.

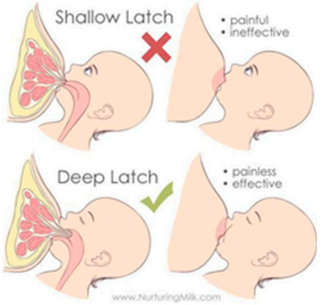

Shallow Latch vs. Deep Latch. Source: Nurturing Milk

When the latch is deep, your baby takes in a generous mouthful of breast tissue, protecting your nipple from friction and letting milk flow freely.

How to Get a Pain-Free Deep Latch

If a latch feels like a pinch, bite, or sharp sting, break the suction gently by slipping a clean finger into the corner of your baby's mouth, and try again using these steps:

1.Aim nipple to nose: Positioning.

Hold your baby tummy-to-tummy, level with your breast. Position them so their nose - not their mouth - is directly opposite your nipple. This encourages them to tilt their head back slightly.

2.Tickle the upper lip: Waiting for a wide open mouth.

Gently brush your nipple against your baby's upper lip. Wait patiently until they open their mouth as wide as a big yawn.

3.Bring baby to breast: Asymmetric attachment.

Bring your baby swiftly to your breast **chin first**, resting their lower jaw well below the areola. The nipple should aim up toward the roof of their mouth.

4.Check the lips and comfort: Post-latch check.

Their top and bottom lips should flange outward like fish lips, covering more of the lower dark area (areola) than the top. Feeding should feel like a deep tugging sensation, not a sharp pain.

Soothing & Healing Your Nipples Between Feedings

While you refine the latch, here are the fastest ways to help damaged skin heal:

- **Fresh Breast Milk:** After a feeding, express a few drops of your own milk, gently rub it over your nipples, and let them air dry. Breast milk contains natural antibodies and antibacterial properties that speed up skin healing.
- **Purified Lanolin or Nipple Butter:** Apply a thin layer of 100% medical-grade lanolin or organic nipple balm after air-drying. This keeps the skin supple and prevents scab formation without needing to be washed off before the next feed.
- **Silver Nursing Cups (Silverettes):** Worn inside your bra between feeds, silver cups soothe raw tissue and prevent your clothing or breast pads from sticking to cracks.

	• **Hydrogel Pads:** Cooling gel pads placed directly over the nipples between feeds provide immediate pain relief and promote moist tissue recovery.<br>• **Change Positions:** Try the **football hold** or **laid-back breastfeeding** position. Changing angles shifts pressure away from sore spots.<br>**When to seek hands-on help:** If pain persists even with a wide latch, if you notice deep cracks, bleeding, or shooting pains deep in the breast, reach out to an **International Board Certified Lactation Consultant (IBCLC)** or your healthcare provider. They can check for underlying issues like baby tongue-tie or thrush (a fungal infection) and help you feed pain-free.
**02. "Our pediatrician told us that the baby is not gaining enough weight and recommended that I start giving formula. However, I really want to continue exclusive breastfeeding. I don't know what to do, and I'm afraid of making the wrong decision. How should I proceed?"**	
**Clinic Response**	During the initial telephone consultation, the mother's concerns were acknowledged, and the available information regarding the infant's feeding and weight gain was reviewed. She was advised that exclusive breastfeeding could be continued provided that the infant demonstrated appropriate weight gain and remained clinically well. Practical, individualized guidance was offered to support effective breastfeeding and adequate milk transfer, and the mother was encouraged to contact the clinic again to report the infant's progress and subsequent weight measurements.<br>The mother called again the following day and reported that the paediatrician continued to recommend formula supplementation. In response, she was invited to bring the infant to the hospital for direct clinical assessment. Examination and weight evaluation confirmed that the infant was clinically well and gaining weight appropriately while being exclusively breastfed. The mother was therefore reassured and advised to continue exclusive breastfeeding with appropriate monitoring. The clinical team subsequently communicated with the paediatrician to establish a coordinated and consistent management plan and to avoid further conflicting advice. This follow-up transformed the initial telephone guidance into direct clinical assessment and coordinated care.
**ChatGPT 5.5**<br>*(on 26-07-2026)*	It's completely understandable to feel conflicted. Wanting to continue exclusive breastfeeding while also wanting your baby to grow well are both valid goals. The good news is that, in many cases, it's possible to protect breastfeeding while also addressing poor weight gain-but the first step is to understand *why* your baby isn't gaining enough weight.<br>Here is a practical approach:<br>**1. Confirm that weight gain is truly inadequate**<br>Ask your pediatrician:<br>• How much weight has your baby gained since the last visit?<br>• How does that compare with the expected gain for your baby's age?<br>• Is the concern based on one measurement or a trend over several weeks?

Sometimes babies simply follow a lower growth percentile, while other times there is genuine insufficient weight gain that needs prompt attention.

2. Evaluate breastfeeding before assuming low milk supply

Many babies who gain weight slowly are **not** affected by low milk production but by ineffective milk transfer. A lactation consultant (IBCLC) can assess:

- Positioning and latch.
- Whether your baby is swallowing milk effectively.
- A feeding session with a pre- and post-feed weight ("weighted feed") to estimate how much milk your baby actually transfers.

3. Optimize breastfeeding

If your baby is otherwise healthy, you may be advised to:

- Breastfeed at least 8–12 times in 24 hours.
- Offer both breasts at each feeding.
- Allow your baby to actively feed before switching sides.
- Use breast compressions when sucking slows.
- Avoid long stretches between feeds, especially in young infants.

4. Protect your milk supply

If supplementation becomes necessary:

- Whenever possible, pump after feeds to stimulate milk production.
- Expressed breast milk is usually the first choice if enough is available.
- Formula can be used when needed while continuing to breastfeed.

Needing temporary supplementation does **not** mean breastfeeding has failed. Many families later return to exclusive breastfeeding once milk supply or feeding effectiveness improves.

5. Follow your baby's weight closely

Your pediatrician may recommend frequent weight checks (for example, every few days or weekly) to ensure your baby's growth improves and to adjust the feeding plan if needed.

Should you give formula?

If your pediatrician believes your baby is not receiving enough nutrition, it is important **not to delay treatment** while investigating the cause. Adequate nutrition is the priority because insufficient intake can affect growth and hydration.

At the same time, introducing formula does **not** necessarily mean you must stop breastfeeding. With appropriate support and a plan to maintain or increase your milk supply, many mothers continue breastfeeding successfully.

Seek additional breastfeeding support

If possible, arrange an appointment with an **International Board Certified Lactation Consultant (IBCLC)** as soon as possible. They can often identify correctable issues that are difficult to detect during a routine pediatric visit.

I have a few questions that will help me give more specific guidance:

- How old is your baby?

	• What was your baby's birth weight, lowest weight after birth, and current weight?<br>• How many wet diapers and dirty diapers does your baby have in 24 hours?<br>• Is your baby exclusively breastfed now, or have you already started giving some formula?
**Gemini 3.5 Flash** *(on 26-07-2026)*	It is completely natural to feel overwhelmed and conflicted right now. Wanting to protect your breastfeeding journey while ensuring your baby stays healthy and thrives is a tough position to be in, but these two goals do not have to be in conflict. Formula supplementation does not mean your breastfeeding relationship is over, and taking immediate steps can help you get clarity on what is happening.<br>Here is a clear, step-by-step path forward to ensure your baby is safe while preserving your breastfeeding goals.<br>**1. Schedule an Appointment with an IBCLC Immediately**<br>While pediatricians are experts in overall child medical care and growth curves, an **International Board Certified Lactation Consultant (IBCLC)** specializes in the mechanics of breastfeeding.<br>An IBCLC can perform a **weighted feed**-weighing your baby before and immediately after nursing on a precise scale-to measure exactly how much milk your baby is transferring per session. They will also evaluate:<br>• **Latch and position:** Ensuring the baby is effectively extracting milk without exhausting themselves.<br>• **Oral anatomy:** Checking for tongue or lip ties that might restrict movement.<br>• **Feed efficiency:** Identifying whether the baby is actively drinking or mostly flutter-nursing.<br>**2. Treat Supplementation as a Temporary Bridge, Not a Final Outcome**<br>If your pediatrician feels supplementation is urgently necessary for safety, remember that supplementation is often a **temporary medical tool to restore energy**, giving your baby the strength to nurse effectively.<br>If you need to supplement in the short term, you have choices:<br>• **Expressed Breast Milk First:** If you can pump after nursing sessions, offered breast milk can be used as the primary supplement before turning to formula.<br>• **Preserving the Breast Connection:** You can use a **Supplemental Nursing System (SNS)**-a thin tube placed next to your nipple while nursing-so your baby receives extra milk at the breast while stimulating your supply. Alternative methods like **paced bottle feeding** or **cup feeding** also prevent flow preference.<br>• **Protecting Your Milk Supply:** Any time your baby receives a bottle or extra supplement, perform a full pumping session (triple feeding) to signal to your body that milk is still required.<br>**3. Partner with Your Pediatrician on a Measurable Plan**<br>You do not have to choose between your doctor's advice and your feeding goals. Communicate openly with your pediatrician about your desire to continue breastfeeding while working on weight gain.<br>Ask your pediatrician:

	• *"Can we set up a weight check in 3 to 7 days to evaluate progress?"*<br>• *"What specific growth target or daily weight gain are we looking for?"*<br>• *"Are you open to me working with a lactation consultant to optimize transfer before making long-term feeding changes?"*<br>Your baby's health and your peace of mind come first, and getting a full professional picture from both a doctor and a lactation specialist will give you the confidence to make the right choice for your family.
**03. "Please, I need your help. My baby is 10 days old, and sometimes when I breastfeed, I experience an orgasm. I feel extremely embarrassed and overwhelmed with guilt. I'm thinking about stopping breastfeeding, but my husband and my mother-in-law insist that I continue, and I can't tell them what is happening to me. Is this normal? Has this happened to other women as well? What should I do?"**	
**Clinic Response**	The mother was reassured that other breastfeeding women had described similar experiences and that an involuntary response of this kind did not imply inappropriate intent or harm to her infant. The consultation explored the frequency of the episodes, the infant's breastfeeding pattern, and the extent of the mother's emotional distress. She was offered nonjudgmental emotional support and encouraged not to blame herself or feel guilty. Continuing direct breastfeeding was supported if she felt comfortable doing so, while temporary milk expression using a breast pump was discussed as an alternative if breastfeeding became too distressing. Given her intense embarrassment and guilt, psychological support was offered and an appointment was arranged.
**ChatGPT 5.5**<br>*(on 26-07-2026)*	First, I want to reassure you of something very important: **what you're experiencing has been described by other breastfeeding women, and it does not mean there is anything wrong with you.** While it is not commonly discussed, it is a recognized phenomenon.<br>Breastfeeding triggers the release of several hormones, especially **oxytocin**, which is responsible for the milk ejection ("let-down") reflex. Oxytocin is also released during labor, skin-to-skin contact, hugging, and sexual activity. Because the same nerves and hormones are involved, **some women experience sensations ranging from warmth and relaxation to, more rarely, sexual arousal or even orgasm during breastfeeding.** This is an involuntary physiological response-it is **not a reflection of your feelings toward your baby or of your intentions.**<br>The fact that you feel embarrassed or guilty is understandable because our society rarely talks about this. However:<br>• **You are not harming your baby.**<br>• **You are not doing anything inappropriate.**<br>• **This does not mean you are confusing breastfeeding with sexuality.**<br>• **You are not alone.** There are published medical reports and personal accounts from other mothers describing the same experience.<br>**Will it go away?**<br>For many women, these sensations become much less intense over time, especially as:<br>• breastfeeding becomes established,<br>• hormone levels stabilize in the weeks after birth,

- the emotional intensity of the early postpartum period decreases.

Since your baby is only **10 days old**, your body is still going through enormous hormonal changes.

Should you stop breastfeeding?

Based solely on what you've described, **there is no medical reason to stop breastfeeding because of these involuntary sensations.** If breastfeeding is otherwise going well and you wish to continue, it is reasonable to do so.

However, your own well-being matters too. If the experience becomes so distressing that it causes severe anxiety, panic, or makes breastfeeding unbearable, it would be worthwhile to speak confidentially with:

- a lactation consultant,
- your obstetrician or midwife,
- or a mental health professional experienced in perinatal care.

They should respond without judgment. Health professionals who work with breastfeeding are generally aware that breastfeeding can evoke a wide range of emotional and physical responses.

Do you have to tell your husband or mother-in-law?

No. You are under **no obligation** to share something so personal if you are not comfortable doing so. If you simply want support in continuing breastfeeding, you can seek it from a healthcare professional instead.

Some things that may reduce the intensity

Some mothers report that it helps to:

- focus on their baby's face and breathing,
- practice slow breathing during let-down,
- change breastfeeding positions,
- reduce external stimulation or distractions,
- remember that the sensation is a temporary bodily reflex rather than something they are choosing.

One more thing

You mentioned feeling "**extremely embarrassed and overwhelmed with guilt.**" Those feelings sound much more painful than the physical sensation itself. From a medical perspective, **there is nothing to feel guilty about.** An involuntary orgasm is a reflex of your nervous and hormonal systems. We generally do not judge ourselves for involuntary yawning, sneezing, or crying because they are not under conscious control. This is different in sensation, but the principle is the same: **your body is producing a response that you did not choose.**

If I may ask one question: **does the orgasm occur only during the first minute or two when your milk lets down, or does it continue throughout the entire feeding?** That detail can help distinguish whether it is most likely related to the oxytocin-driven let-down reflex, which is the pattern most commonly described.

Gemini 3.5 Flash *(on 26-07-2026)*

Please take a deep breath: **Yes, this is completely normal, it has happened to many other women, and you have no reason to feel guilty.** You are not broken, wrong, or a bad mother.

What you are experiencing is a **purely physical, biological reflex**, not a sexual or psychological desire toward your newborn. You do not need to stop breastfeeding, nor do you need to confess this to your family if you do not feel comfortable doing so.

Here is why your body is reacting this way and what you can do to manage it.

Why Is This Happening?

Breastfeeding and sexual arousal rely on the exact same anatomical pathways and hormones. Your body cannot always distinguish between nipple stimulation from nursing and nipple stimulation from intimate touch.

- **The Oxytocin Surge:** As your baby suckles, your brain releases massive surges of **oxytocin** (the hormone responsible for milk let-down). Oxytocin is the exact same hormone released during physical intimacy and orgasm.
- **Uterine Contractions:** In the first few weeks postpartum (especially around day 10), oxytocin causes your uterus to contract back to its pre-pregnancy size. These pelvic floor and uterine contractions directly mimic the physical sensation of an orgasm.
- **Nerve Stimulation:** The nipples are dense with nerve endings. Continuous, strong suckling from a newborn triggers reflex responses in the pelvic nerves, which can automatically trigger an involuntary climax.
- **Increased Blood Flow:** Postpartum blood flow to the pelvic region remains extremely high, making the pelvic area extra sensitive.

This is a **mechanical and hormonal response**-much like blinking when something flies toward your eye or laughing when you are tickled. Your body is simply functioning as it was wired biologically.

What Should You Do?

1. **Separate the Reflex from Your Intentions**

Recognize that an involuntary nerve response does not reflect your thoughts, morals, or feelings as a mother. Remind yourself: *"This is just a biological reflex from oxytocin and nerve stimulation."*

2. **Adjust Your Posture**

Pay attention to how you sit while feeding. Crossing your legs or tilting your pelvis forward can press your body or clothing against your clitoral area, increasing physical stimulation. Sitting upright with your feet flat on the floor or reclining slightly can reduce pelvic pressure.

3. **Change the Environment**

If you feel an intense reaction coming on, try shifting your focus. Keep the TV on, listen to a podcast, or talk out loud to distract your nervous system from the sensory input.

4. **Give It Time**

As your uterus finishes shrinking over the next few weeks and your hormone levels stabilize, these intense oxytocin spikes and involuntary pelvic contractions usually calm down significantly.

5. **Keep Your Privacy**

You do not owe anyone an explanation. You do not need to share this with your husband or mother-in-law if it causes you distress. If you need professional support, consider reaching out to an International

	Board Certified Lactation Consultant (IBCLC) or a postpartum therapist-healthcare professionals are very familiar with this phenomenon and will handle it with discretion and zero judgment. You are doing a wonderful job caring for your 10-day-old baby. Give yourself grace as your body navigates the overwhelming changes of the early postpartum period.
**04. "My baby is unable to latch onto my nipple, and so far I have been expressing my milk by hand to feed the baby. However, I am completely exhausted and am thinking about stopping breastfeeding. How can I wean safely? Is there any other solution?"**	
**Clinic Response**	During the consultation, the mother received individualized guidance on positioning the infant at the breast, achieving effective attachment, and trying alternative breastfeeding positions to facilitate latching. Because repeated hand expression had become physically exhausting, the use of a breast pump was recommended as a more practical method of maintaining milk expression while continuing efforts to improve attachment.<br>The mother then disclosed that financial difficulties prevented her from purchasing a breast pump. In response, the midwife contacted a breast pump company collaborating with the hospital and arranged for the mother to receive a pump free of charge. During follow-up communication, the mother confirmed that she was using the pump successfully and was able to continue providing her infant with expressed breast milk.
**ChatGPT 5.5** *(on 26-07-2026)*	I'm sorry you're going through this. What you're describing is very common, especially after a difficult start to breastfeeding. Expressing milk by hand or pumping around the clock while trying to care for a newborn is physically and emotionally exhausting. If you're feeling ready to stop, that's a valid choice. If your goal is to continue providing breast milk, there may still be options that don't require you to stop immediately.<br>**Before deciding to wean, consider whether the latch problem can still be solved**<br>Many babies who initially refuse the breast-especially if they were premature, separated from their mother, or mainly fed by bottle-can learn to latch later with support.<br>You could try:<br>• Spending lots of skin-to-skin time with your baby, without pressure to feed.<br>• Offering the breast when your baby is sleepy or just waking up, when they're often calmer.<br>• Having an experienced lactation consultant observe a full feeding to identify problems such as positioning, tongue-tie, or ineffective sucking.<br>• Using techniques such as paced bottle feeding if bottles are being used, which can make returning to the breast easier.<br>• In some situations, a nipple shield or a supplemental nursing system may help, but these are best used with professional guidance.<br>Even if your baby eventually breastfeeds only some of the time, that can reduce the amount of expressing you need to do.

	**If you decide to stop breastfeeding, wean gradually**<br>Stopping suddenly can cause painful breast engorgement, blocked ducts, or mastitis.<br>A gradual approach is usually safest:<br>1. Drop one expressing session every 2–3 days.<br>2. Shorten the remaining sessions so you express only enough to relieve discomfort rather than fully emptying the breasts.<br>3. Wear a comfortable, supportive bra (not one that is excessively tight).<br>4. Apply cool compresses between expressing sessions if your breasts feel swollen.<br>5. Use pain relief such as ibuprofen or acetaminophen if they're appropriate for you, following the directions on the label or your healthcare professional's advice.<br>6. Stay hydrated-you don't need to restrict fluids.<br>Milk production generally decreases over several days to a few weeks, depending on how much milk you're currently making.<br>**Seek medical advice promptly if you develop:**<br>• A fever (100.4°F/38°C or higher)<br>• A painful, red, hot area on the breast<br>• Flu-like symptoms<br>• Severe breast pain that doesn't improve after expressing a small amount of milk<br>These can be signs of mastitis, which may need treatment.<br>**It's also okay to choose a middle ground**<br>You don't have to choose between "exclusive breastfeeding" and "completely stopping." Some parents find that:<br>• They breastfeed when possible and use formula for the remaining feeds.<br>• They express less often and gradually transition.<br>• They continue one or two breastfeeds a day for comfort and bonding.<br>Any amount of breast milk your baby receives has value, and your wellbeing matters too.<br>If you're willing, could you tell me:<br>• **How old is your baby?**<br>• **Was your baby born prematurely or admitted to the NICU?**<br>• **Are you expressing by hand only, or are you also using a breast pump?**<br>Those details can help determine whether improving the latch is still a realistic option or whether a gradual weaning plan is likely to be the best fit.
**Gemini 3.5 Flash** *(on 26-07-2026)*	It is completely understandable that you feel exhausted. Hand expressing exclusively takes a massive amount of physical and mental energy on top of newborn sleep deprivation. First, give yourself grace: **a fed baby and a healthy, mentally present mother are what matter most**, regardless of how your baby gets fed.<br>Whether you decide to pivot to a different strategy or step away from breast milk entirely, you have options.<br>**1. Options if You Want to Keep Trying**

	If you still hope to provide breast milk without relying on the physical toll of hand expressing, consider these alternatives:<br>● **Consult an IBCLC:** An International Board Certified Lactation Consultant can examine your baby's mouth for physical obstacles (such as a tongue-tie) and adjust your positioning.<br>● **Try a Silicone Nipple Shield:** A thin, flexible silicone cover placed over your nipple gives the baby a firmer surface to grip. It often bridges the gap for babies struggling with direct contact.<br>● **Switch to an Electric Breast Pump:** Hand expressing every single meal is grueling work. A double electric breast pump automates the process and frees up significant time and effort.<br>● **Combination Feeding:** You do not have to pick all or nothing. You can substitute some feeds with formula to give yourself time to rest while keeping a lower-stress breast milk routine.<br>**2. How to Wean Safely**<br>If you have decided to stop, it is essential to **wean gradually** to avoid complications like painful engorgement, blocked milk ducts, or mastitis.<br>**The Phase-Out Process**<br>1. **Reduce Hand Expressing Gradually:** Do not stop cold turkey. Slow down step-by-step.<br>2. **Express Only for Comfort:** When your breasts feel full or uncomfortable, hand express *just enough* to relieve the pressure-never to empty the breast. Fully emptying the breast signals your body to keep producing milk.<br>3. **Space Out Sessions:** Stretch the time between expressing sessions longer and longer each day.<br>**Managing Comfort & Symptoms**<br>● **Cold Compresses:** Apply ice packs or cold gel packs for 10–15 minutes at a time to ease swelling and inflammation.<br>● **Supportive Bra:** Wear a well-fitting, supportive bra without an underwire. Avoid tight binding, which can lead to clogged ducts.<br>● **Pain Relief:** Over-the-counter anti-inflammatories (like ibuprofen) can help manage swelling and pain.<br>⚠ **When to seek medical attention:** Watch out for signs of **mastitis**. If you develop a hard, painful red lump, severe breast pain, or flu-like symptoms (chills, fever, body aches), contact a doctor right away.
**05. "I hate having someone touch my breasts, but I support breastfeeding. I would like to breastfeed my baby, but I can't stand the sensation. What can I do?"**	
**Clinic Response**	The mother's discomfort was acknowledged without judgment, and she was reassured that supporting breastfeeding does not mean that she must tolerate physical contact that causes significant distress. Targeted questions were used to explore whether the aversion was limited to direct breastfeeding or also occurred with hand expression and breast-pump use, and whether it was associated with pain, anxiety, previous negative experiences, or other distressing sensations.
**ChatGPT 5.5** *(on 26-07-2026)*	Many people are surprised to discover that they have a very strong aversion to having their breasts or nipples touched, even if they genuinely want to breastfeed. This is more common than many realize, and it does **not** mean you are a bad parent or that you won't be able to breastfeed successfully.

	Several things may help:<br>● **Try to identify what is most difficult.** Is it the physical sensation of nipple stimulation? Pain? A feeling of being "touched out"? Anxiety? Knowing what triggers the distress can guide solutions.<br>● **Optimize the latch and positioning.** A deep, comfortable latch can reduce unpleasant sensations. An International Board Certified Lactation Consultant (IBCLC) can assess positioning and help make feeds more comfortable.<br>● **Reduce sensory overload.** Some parents find it easier to breastfeed in a quiet, dimly lit room while listening to music, practicing slow breathing, holding a soft blanket, or focusing on another sensory input.<br>● **Feed before becoming overwhelmed.** If the baby is extremely hungry and suckles vigorously, the sensation may be more intense. Offering the breast earlier may help.<br>● **Use gradual exposure.** Some parents find that the discomfort lessens over days or weeks as breastfeeding becomes more familiar. There is no need to force yourself beyond what feels manageable.<br>● **Consider expressing milk temporarily.** If direct breastfeeding feels unbearable, pumping or hand expression can provide breast milk while you work on becoming more comfortable with nursing. You can also combine expressed milk with direct breastfeeding if that works better for you.<br>It's also worth considering whether you have experienced **breast or nipple sensitivity before pregnancy**, or whether there is a history of trauma or sensory processing differences. If so, discussing this with a healthcare professional, therapist, or lactation consultant can be very helpful. They can suggest individualized strategies that respect your comfort and boundaries.<br>Most importantly, **you do not have to endure severe distress to be a good parent**. Breastfeeding should not come at the expense of your mental well-being. If, after trying different approaches and receiving support, breastfeeding remains intolerable, there are other safe ways to feed and bond with your baby.<br>May I ask: **is the sensation mainly physical (it feels unbearable on your nipples), emotional (you feel distressed or violated), or both?**
**Gemini 3.5 Flash** *(on 26-07-2026)*	It is completely understandable to feel torn when your physical comfort conflicts with how you want to feed your baby. What you're experiencing is actually far more common than most people realize, and wanting to advocate for your baby's nutrition while protecting your personal boundaries is entirely valid.<br>There are several biological, psychological, and sensory reasons why touch in this area can feel intolerable:<br>● **Sensory Processing or Tactile Aversion:** Heightened sensitivity to physical touch, pressure, or temperature shifts can make breast contact feel overwhelming or invasive.<br>● **Dysmorphic Milk Ejection Reflex (D-MER):** This is a physiological condition where a sudden drop in dopamine right before milk release causes brief waves of intense aversion, anxiety, or unease during touching or feeding.

- **Past Trauma or Body Boundaries:** For many parents, breast contact brings up complex emotional or physical boundaries that make direct touching distressing.

Practical Approaches to Try

Here are several actionable strategies that can help minimize direct physical touch while still providing breast milk or building a manageable feeding routine:

1. Create a Physical Barrier with Nipple Shields

A silicone **nipple shield** acts as a thin protective barrier between your skin and your baby's mouth, altering the physical texture and reducing direct contact sensation.

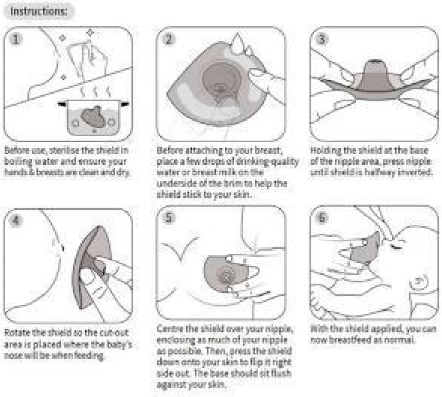

Silicone Nipple Shield Application. Source: Haakaa Middle East

2. Exclusive Pumping (Hands-Free Option)

You do not have to put your baby to the breast to breastfeed. Many parents choose to **exclusively pump**:

- **Hands-free electric pumps** or wearable pump cups fit inside a bra, allowing you to extract milk with zero hands-on contact.
- You can then feed your baby breast milk via a bottle, completely separating the sensation of nursing from the act of feeding.

3. Focus on Visual and Mental Distraction

If you choose to try direct nursing, changing your sensory focus during the feed can help quiet the brain's reaction:

- Use noise-canceling headphones with calming audio or podcasts.
- Hold a fidget toy or ground yourself with a texture you enjoy in your non-holding hand.
- Feed in low-light environments to reduce visual stimulation.

4. Combination Feeding or Formula

Your mental health and physical comfort matter just as much as milk production. You can choose to combo-feed (part breast milk, part formula) to reduce how often you need to express, or switch fully to formula if the sensory discomfort remains overwhelming. **A well-fed baby and a regulated, comfortable parent is always the best outcome.**

A helpful step: Consider speaking with an **IBCLC (International Board Certified Lactation Consultant)** who specializes in sensory aversions or trauma-informed care. They can help you tailor a feeding plan that prioritizes *your* comfort.
